# Characterization of pre- and on-treatment soluble immune mediators and the tumor microenvironment in NSCLC patients receiving PD-1/L1 inhibitor monotherapy

**DOI:** 10.1007/s00262-024-03781-8

**Published:** 2024-09-05

**Authors:** Daiki Murata, Koichi Azuma, Kenta Murotani, Akihiko Kawahara, Yuuya Nishii, Takaaki Tokito, Tetsuro Sasada, Tomoaki Hoshino

**Affiliations:** 1 Division of Respirology, Neurology, and Rheumatology, Department of Internal Medicine, Kurume University School of Medicine, 67 Asahi-Machi, Kurume, Fukuoka, 830-0011 Japan; 2 Biostatistics Center, Kurume University School of Medicine, Fukuoka, Japan; 3 Department of Diagnostic Pathology, Kurume University School of Medicine, Fukuoka, Japan; 4 Cancer Vaccine and Immunotherapy Center and Division of Cancer Immunotherapy, Kanagawa Cancer Center Research Institute, Kanagawa, Japan

**Keywords:** NSCLC, Immune checkpoint inhibitor, Chemokine, Cytokine, CD8+ TILs, Biomarker

## Abstract

**Background:**

Despite the favorable therapeutic efficacy observed with ICI monotherapy, the majority of non-small cell lung cancer (NSCLC) patients do not respond. Therefore, identifying patients who could optimally benefit from ICI treatment remains a challenge.

**Methods:**

Among 183 patients with advanced or recurrent NSCLC who received ICI monotherapy, we analyzed 110 patients whose pre- and post-treatment plasma samples were available. Seventy-three soluble immune mediators were measured at ICI initiation and 6 weeks later. To identify useful biomarkers, we analyzed the association of pre-treatment levels and on-treatment changes of soluble immune mediators with survival of patients. The associations of pre-treatment or on-treatment biomarkers with irAE development, PD-L1 expression, CD8+ TIL density, and neutrophil to lymphocyte ratio (NLR) were also analyzed.

**Results:**

Univariate analysis showed that pre-treatment biomarkers included 6 immune mediators, whereas on-treatment biomarkers included 8 immune mediators. Multivariate analysis showed that pre-treatment biomarkers included 4 immune mediators (CCL19, CCL21, CXCL5, CXCL10), whereas on-treatment biomarkers included 5 immune mediators (CCL7, CCL19, CCL23, CCL25, IL-32). IrAE development was associated with on-treatment change in CCL23. PD-L1 expression was associated with the pre-treatment levels of TNFSF13B and the on-treatment change in CCL25. CD8+ TIL density was associated with the pre-treatment CXCL10 level, whereas NLR was correlated with pre-treatment levels of CCL13 and CCL17.

**Conclusion:**

We identified several soluble immune mediators as pre-treatment and on-treatment biomarkers of survival in patients with NSCLC treated with ICI monotherapy. Some of these biomarkers were associated with other possible predictors, including irAE development, PD-L1 expression, CD8+ TIL density and NLR. Further large-scale studies are needed to establish biomarkers for patients with NSCLC who received ICI monotherapy.

**Supplementary Information:**

The online version contains supplementary material available at 10.1007/s00262-024-03781-8.

## Introduction

Immune checkpoint inhibitors (ICI) have become the new standard of treatment for advanced and recurrent non-small cell lung cancer (NSCLC). Inhibitors of programmed cell death-1 (PD-1)/programmed cell death ligand-1 (PD-L1) and cytotoxic T lymphocyte antigen 4 (CTLA-4) activate tumor-specific T cells and provide therapeutic efficacy. These agents are characterized by long-term survival and sustained therapeutic efficacy even after discontinuation of treatment. Despite the favorable therapeutic efficacy observed, the majority of patients do not respond to ICI monotherapy. Therefore, a current challenge is to identify those patients who could optimally benefit from ICI treatment [1–6].

The cancer-immunity cycle does not function optimally in advanced cancer patients. For an antitumor immune response to result in the effective killing of tumor cells, a series of stepwise events must be initiated and allowed to proceed and expand iteratively. This cycle can be divided into seven major steps, beginning with the release of antigens from tumor cells and ending with the killing of tumor cells. Each step of the cancer-immunity cycle requires the coordination of numerous factors, both stimulatory and inhibitory in nature [1]. Soluble immune mediators, including cytokines and chemokines such as those in the interleukin (IL) family, the tumor necrosis factor superfamily (TNFSF), chemokine ligands (CCL), and C-X-C motif chemokine ligands (CXCL), can stimulate or inhibit each step of the cycle [1, 2, 7–15]. The tumor microenvironment (TME) forms a complex network of cytokines or chemokines that modulate antitumor immunity. While a tremendous amount of research has been conducted on cancer immunology and immunotherapy to implement clinical strategies, the role of various immune cells in the TME remains unclear [1–3, 6–15].

In addition to characterizing soluble immune mediators, each step of the cancer immune cycle can be assessed by immunohistochemical analysis. PD-L1 expression in tumor tissue suggests inhibition of the step of cancer cell killing. The density of CD8+ T cells in tumor tissue indicates the ability of T cells to infiltrate into the tumor [1–3]. The TME has been divided into four different types based on PD-L1 expression and CD8+ tumor-infiltrating lymphocytes (TILs); type I (PD-L1 positive with TILs driving adaptive immune resistance), type II (PD-L1 negative with no TIL indicating immune ignorance), type III (PD-L1 positive with no TIL indicating intrinsic induction), and type IV (PD-L1 negative with TIL indicating the role of other suppressor in promoting immune tolerance) [3]. This classification provides a framework for predicting the therapeutic outcome of cancer immunotherapy. However, since tissue biopsies are invasive and time-consuming, simpler non-invasive methods are needed.

A comprehensive study of biomarkers such as cytokines and chemokines may lead to a better understanding of the relationship between cancer immunity and immunotherapy and may identify novel therapeutic targets [1–3, 7–15]. Therefore, we measured a comprehensive set of soluble immune mediators and performed an exploratory analysis in patients with NSCLC who received PD-1/L1 monotherapy. In the present study, 73 soluble immune mediators were measured at ICI initiation and 6 weeks later. We analyzed the association of patient survival and the levels of soluble immune mediators at ICI initiation as a pre-treatment biomarker. We also analyzed the association between patient survival and the changes in soluble immune mediators 6 weeks after ICI initiation as an on-treatment biomarker, as it may reflect the changes in the TME that are associated with treatment efficacy. The correlation between pre-treatment and on-treatment biomarkers was examined to investigate the dynamics of soluble immune mediators in ICI treatment. The correlations between pre-treatment or on-treatment biomarkers and other possible predictors, including the development of immune-related adverse events (irAE), PD-L1 expression, CD8+ TIL density, peripheral blood cells and the neutrophil to lymphocyte ratio (NLR) were examined to reveal the underlying mechanisms associated with therapeutic outcome.

## Materials and methods

### Study design

We retrospectively screened patients with advanced or recurrent NSCLC who received PD-1/L1 monotherapy at Kurume University Hospital between January 2016 and December 2020. As a result of screening, there were 183 patients pathologically diagnosed NSCLC and treated with PD-1/L1 monotherapy. Among 183 patients, 73 were excluded because pre- and/or post-treatment plasma samples were not available, and 110 patients were analyzed. Plasma samples were collected at ICI initiation and 6 weeks later. Nivolumab was administered every 2 weeks, and pembrolizumab and atezolizumab were every 3 weeks. EGFR mutations and ALK rearrangements were tested by single or multi-plex and negative patients were classified as wild type. Progression-free survival (PFS) and overall survival (OS) were calculated for each patient. This study was conducted in accordance with the provisions of the Declaration of Helsinki and was approved by the Institutional Review Board of the Kurume University Hospital (IRB No 20100).

### Measurement of soluble immune mediators in plasma

We collected plasma samples at the time of ICI initiation and 6 weeks later. Since there is no established time to evaluate the blood sample after ICI treatment initiation, we set it at 6 weeks later based on the time of tumor assessment in clinical trials and previous studies [6, 13]. Radiological evaluations were also performed at the same time.

**Fig. 1:**
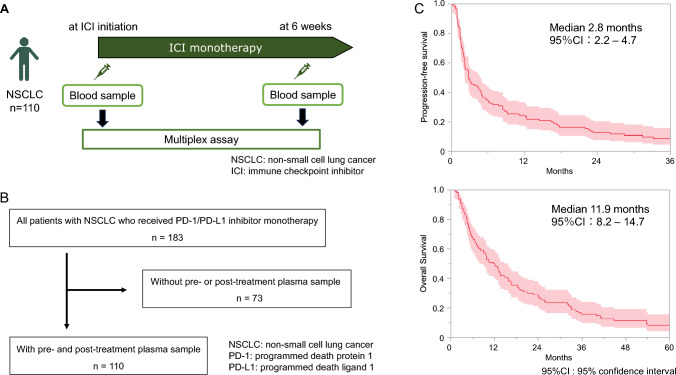
**A** Overview of the study. We analyzed 110 patients for whom pre- and post-treatment plasma samples were available. Plasma samples were collected at ICI initiation and 6 weeks later. A multiplex assay was used to measure plasma levels of 73 soluble immune mediators. The associations between the 73 immune mediators and patient prognosis were analyzed. **B** Flow diagram of the study population. Among the 183 patients with advanced or recurrent NSCLC who received ICI monotherapy, we analyzed 110 patients whose pre- and post-treatment plasma samples were available. **C** Kaplan–Meier survival curves for PFS and OS. The median PFS was 2.8 months (95%CI: 2.2–4.7), and the median OS was 11.9 months (95%CI: 8.2–14.7). *95%CI: 95% confidence interval, ICI: immune checkpoint inhibitor, PFS: progression-free survival, OS: overall survival*

This was done in order to explore biomarkers associated with prognosis of NSCLC patients (Fig. 1A). All samples were heparinized and centrifuged at 1600 g for 15 min. The plasma supernatants were transferred to new tubes and stored at –80 °C until measurement. At the time of measurement, these samples were allowed to thaw naturally at room temperature. Soluble immune mediators were measured once for each patient and measurements were not repeated. A bead-based multiplex assay was used to measure plasma levels of soluble immune mediators. On-treatment changes were calculated as the difference in soluble immune mediator levels found at ICI initiation and 6 weeks later.

A Bio-Plex 200 system (Bio-Rad Laboratories, Hercules, CA) was used to analyze 100-µL aliquots of two-fold diluted plasma samples in accordance with the manufacturer’s instructions. Kits (Bio-Rad Laboratories) for the various analytes were used to measure the 73 soluble immune mediators, including cytokines, chemokines and growth factors (Supplemental appendix). These soluble immune mediators were selected for their relevance to the TME and the cancer immune cycle [1, 6–15].

### Selection of other possible predictors

We also analyzed the association and correlation between possible predictors of the therapeutic efficacy of ICI monotherapy and pre- and on-treatment biomarkers. Based on previous reports, irAE development, PD-L1 expression, CD8+ TIL density, peripheral blood cell count and proportion, and NLR were selected for examination [1–3, 16–20].

### Definition of irAE, peripheral blood cell, and NLR

IrAEs were evaluated according to the National Cancer Institute Common Terminology Criteria for Adverse Events, version 4.0. Peripheral blood cell counts and proportions were commercially assayed. The data included absolute counts and the proportions of neutrophils, lymphocytes, monocytes, and eosinophils. The NLR was also assessed and calculated using absolute counts of neutrophils and lymphocytes [19]. On-treatment changes in peripheral blood cells were calculated as the difference between the values at ICI initiation and those 6 weeks later.

### Immunohistochemical analysis

In this study, immunohistochemical analysis of PD-L1 expression and CD8+ TIL density was performed in patients whose tumor biopsy specimens were available. All tumor biopsy specimens were taken prior to ICI initiation. In patients with multiple biopsies, specimens closer to ICI initiation were utilized. Four-mm-thick sections of formalin-fixed, paraffin-embedded tissues were used.

The sections were mounted on glass slides and then incubated with anti-rabbit monoclonal antibody against PD-L1 (clone ELL) (Cell signaling Technology, Denver) for immunohistochemical (IHC) analysis using the BenchMark ULTRA (Ventana Automated Systems, Inc., Tucson, AZ, United States of America). Each slide was heat-treated with Ventana’s CC1 retrieval solution for 30 min and incubated with the PD-L1 antibody for 30 min. This automated system used the ultraVIEW DAB detection kit with 3, 3ʹ diaminobenzidine (DAB) as the chromogen (Ventana Automated Systems). PD-L1 expression was categorized as either < 1% or > 1%.

Immunostaining for CD8 (Leica Microsystems, Newcastle-upon-Tyne, UK) was performed on the same fully automated Bond-III system (Leica Microsystems) using on-board heat-induced antigen retrieval with epitope retrieval solution 2 for 10 min at 99 °C, and incubated with the antibody for 30 min at room temperature. This automated system used a Refine polymer detection kit with horseradish peroxidase-polymer as the secondary antibody and DAB, and incubation with a secondary antibody was performed for 30 min at room temperature. TILs were counted on immuno-stained CD8 preparations in randomly selected four representative samples within the tumor area, and the averages were assessed. Necrotic tumor areas were excluded from this assessment, and representative areas were designed to avoid overlap.

### Statistical analysis

This investigation was an observational study. Thus, the target sample size for this study was set at 100 participants. Comparisons of categorical variables were evaluated using chi-squared or Fisher’s exact tests. PFS was defined as the period from the date of the first dose to the date of disease progression or death due to any cause. OS was defined as the period from the date of the first dose to the date of death from any cause. PFS and OS were compared between groups using a log-rank test. To explore factors associated with the dependent variables (PFS and OS), univariate Cox proportional hazards model analysis was performed using soluble immune mediators as independent variables. Due to the limited number of events for the dependent variables in this study, adjusted analysis was not performed. The optimal cut‐off values for each biomarker were determined using the web application Cutoff Finder [21]. The optimal cut‐off was defined as the value resulting in the most significant split (log‐rank test). Spearman correlation analysis was performed to assess the correlation between pre- and on-treatment biomarkers, pre-treatment biomarkers and pre-treatment peripheral blood cells, and on-treatment biomarkers and on-treatment changes in peripheral blood cells. Wilcoxon rank sum tests were used to analyze the association between pre- or on-treatment biomarkers and irAE development, PD-L1 expression and CD8+ TIL density. All tests utilized a two-sided approach, and differences were considered statistically significant at *p* < 0.05. Statistical analyses were performed using JMP pro version 16.0 statistical software (SAS Institute Inc.). The cut-off date for the analyses was March 31, 2023.

The primary endpoint identified pre-treatment and on-treatment biomarkers that were associated with PFS and OS in patients with NSCLC receiving PD-1/L1 monotherapy. Pre-treatment biomarkers were defined as soluble immune mediators whose levels at ICI initiation were associated with PFS and OS. On-treatment biomarkers were defined as soluble immune mediators whose changes after ICI initiation (levels at 6 weeks after ICI initiation – levels at ICI initiation) were associated with PFS and OS. The secondary endpoint was to examine the correlation between pre-treatment and on-treatment biomarkers. Additional secondary endpoints included the associations of pre-treatment and on-treatment biomarkers with PD-L1 expression or CD8+ TIL density. The correlations between pre-treatment biomarkers and pre-treatment peripheral blood cells and between on-treatment biomarkers and on-treatment changes in peripheral blood cells were also analyzed.

## Results

### Patient characteristics and survival

Here, we focused on patients with NSCLC who had received ICI monotherapy. We collected plasma samples at baseline and after 6 weeks of therapy to investigate biomarkers associated with prognosis (Fig. 1A). Among 183 patients with NSCLC who had received ICI monotherapy, we analyzed 110 patients for whom pre- and post-treatment plasma samples were available. Figure 1B shows a flow chart of the study patients. The characteristics of the enrolled patients and the Cox proportional hazard model for PFS and OS are shown in Table 1. The median age was 72 years. Of the 110 patients, 79 were male and 81 had a history of smoking. Performance status was 0–1 in 87 patients and 2–3 in 23 patients. Non-squamous and squamous cell carcinoma were present in 78 and 32 patients, respectively. A driver mutation was harbored in 23 patients, an epidermal growth factor receptor mutation in 20 and an anaplastic lymphoma kinase fusion gene in 3. PD-L1 expression was evaluable in 93 patients; 30 had < 1% and 63 had > 1% (24 had1-49% and 39 had ≥ 50%). There were 17 patients with unknown PD-L1 expression. ICI was administered as first-line, second-line, and third-line or later treatment in 25, 65, and 20 patients, respectively. ICI types were nivolumab in 61, pembrolizumab in 39, and atezolizumab in 10 patients. Nivolumab was administered once, twice, and three times to 2, 12, and 47 patients, respectively, during 6 weeks after treatment initiation. Pembrolizumab was administered once to 7 and twice to 32, and atezolizumab was administered twice to 10. The median PFS was 2.8 months (95%CI: 2.2–4.7), and the median OS was 11.9 months (95%CI: 8.21–4.7). In a multivariate analysis based on univariate analysis of patient characteristics in the Cox proportional hazard model, PFS and OS were significantly associated with only PS (*p* < 0.001). Kaplan–Meier curves for the study patients and each patient characteristics are shown in Fig. 1C and Supplemental Figs. 1 and 2.

**Table 1:** Patient characteristics

Variables	N = 110
Age (range)	72 (53–89)
Gender	
Male	79
Female	31
Smoking history	
+	81
−	29
Performance status	87/23
0–1	87
2–3	23
Histology	
Squamous	32
Non-Squamous	78
Driver mutation	
EGFR	20
ALK	3
Wild type	87
Treatment line	
1st	25
2nd	65
≥ 3rd	20
Immune checkpoint inhibitor	
Atezolizumab	10
Nivolumab	61
Pembrolizumab	39

*ALK* anaplastic lymphoma kinase fusion, *EGFR* epidermal growth factor receptor

**Fig. 2:**
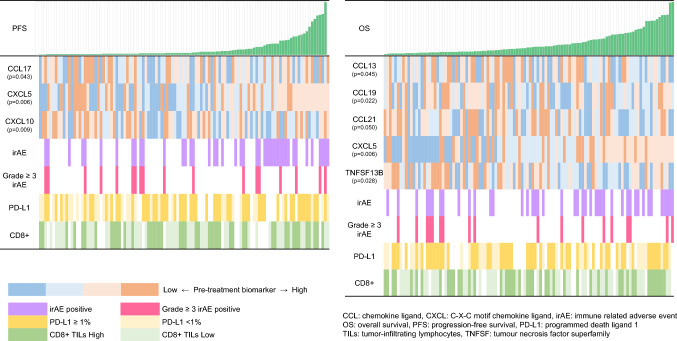
Heatmap showing the associations of patient survival with pre-treatment biomarkers, irAE development, PD-L1 expression and CD8+ TIL density. The *p* values represent the results of univariate analysis for each pre-treatment biomarker. The colors of the pre-treatment biomarkers were classified in a four-tier scale. The cu-toff level was the quartile point for each pre-treatment biomarker. Patients with any grade irAE or Grade > 3 irAE are also colored. PD-L1 expression was categorized as < 1% and > 1% for evaluable patients. CD8+ TIL density was categorized into high and low groups based on median values. For evaluable patients, PD-L1 expression and CD8+ TIL density are shown in two colors. *irAE: immune-related adverse event, PD-L1: programmed cell death ligand-1, TILs: tumor-infiltrating lymphocytes*

### Analysis of pre-treatment or on-treatment biomarkers

We analyzed the association between the levels of soluble immune mediators at ICI initiation and PFS and OS. Among 73 soluble immune mediators, PFS was significantly associated with CXCL5 (*p* = 0.006), CXCL10 (*p* = 0.009), and CCL17 (*p* = 0.043). OS was significantly associated with CXCL5 (p = 0.006), CCL13 (*p* = 0.045), CCL19 (*p* = 0.022), CCL21 (*p* = 0.050) and TNFSF13B (*p* = 0.028). Results of Cox proportion hazard model are shown in Supplemental Table 1A. A heatmap displaying the association between patient survival and pre-treatment biomarkers is shown in Fig. 2. Kaplan–Meier curves for each pre-treatment biomarker are shown in Supplemental Figs. 3 and 4.

**Fig. 3:**
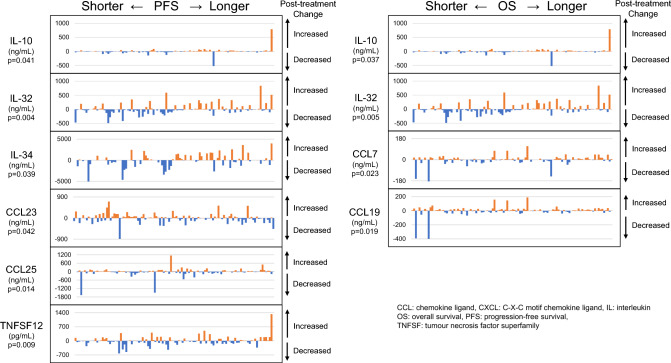
On-treatment changes in soluble immune mediators associated with patient survival. For each patient, increased on-treatment changes are indicated by orange bars and decreased on-treatment changes are indicated by blue bars. The *p* values represent the results of univariate analysis for each on-treatment biomarker

We also analyzed the association between changes in soluble immune mediators 6 weeks after ICI initiation as well as with PFS and OS. Among 73 soluble immune mediators, PFS was significantly associated with CCL23 (*p* = 0.042), CCL25 (*p* = 0.014), IL-10 (*p* = 0.041), IL-32 (*p* = 0.004), IL-34 (*p* = 0.039) and TNFSF12 (*p* = 0.009). OS was significantly associated with CCL7 (*p* = 0.023), CCL19 (*p* = 0.019), IL-10 (*p* = 0.037) and IL-32 (*p* = 0.005). Results of Cox proportion hazard model are shown in Supplemental Table 1B. On-treatment changes in soluble immune mediators associated with patient survival are shown in Fig. 3. Kaplan–Meier curves for each on-treatment biomarker are shown in Supplemental Figs. 5 and 6.

Additionally, we performed a multivariate analysis for each pre-treatment and on-treatment biomarker. This analysis was adjusted for gender (male vs. female), smoking history (current or former smoker vs. never smoker), PS (0–1 vs. 2–3), driver gene mutation (EGFR or ALK vs. wild type), and treatment line (1st line vs. 2nd line or later). Gender and smoking history were selected based on patient characteristics associated with survival, while the other three factors were selected for their clinical importance. For pre-treatment biomarkers, PFS was significantly associated with CXCL5 (*p* < 0.001) and CXCL10 (*p* = 0.008), and OS was significantly associated with CCL19 (*p* = 0.006), CCL21 (*p* = 0.018), CXCL5 (*p* = 0.001). For on-treatment biomarkers, PFS was significantly associated with CCL23 (*p* = 0.021), CCL25 (*p* = 0.003), and IL-32 (*p* = 0.018), and OS was significantly associated with CCL7 (*p* = 0.010), CCL19 (*p* = 0.023), CCL25 (*p* = 0.024), IL-32 (*p* = 0.006). The results of the multivariate analysis are shown in Table 2.

**Table 2:** Association of the levels of pre-treatment biomarkers (A); post-treatment changes in on-treatment biomarkers (B) with irAE development, PD-L1 expression and CD8+ TIL density

Pre-treatment biomarker	irAE development			PD-L1 expression			CD8+ TIL density		
	Positive (N = 43)	Negative (N = 67)	*p* value	Positive (N = 63)	Negative (N = 30)	*p* value	High (N = 44)	Low (N = 45)	*p* value
CCL13	17.6 (14.4–22.2)	19.1 (14.6–26.5)	0.323	19.7 (15.9–26.0)	17.0 (14.5–22.6)	0.207	18.9 (15.3–22.4)	18.5 (14.17–26.37)	0.617
CCL17	10.1 (5.3–18.2)	9.0 (1.1–16.3)	0.273	9.0 (2.7–14.0)	10.1 (5.3–24.3)	0.164	8.5 (1.3–15.6)	9.4 (4.22–17.56)	0.277
CCL19	39.2 (20.6–52.0)	36.3 (18.1–54.4)	0.762	39.2 (19.9–55.4)	35.3 (19.5–49.9)	0.895	33.7 (20.1–48.6)	40.2 (19.4–54.6)	0.458
CCL21	6366.3 (1765.7–8497.4)	6706.2 (3464.6–8730.9)	0.544	6804.1 (5286.5–8811.5)	6366.3 (1054.5–8561.3)	0.073	6762.7 (4166.9–8535.7)	6543.4 (1537.6–8732.8)	0.574
CXLC5	5.7 (5.7–124.4)	5.7 (5.7–52.1)	0.686	5.7 (5.7–51.7)	5.7 (5.7–125.0)	0.259	5.7 (5.7–73.9)	5.7 (5.7–69.5)	0.904
CXCL10	33.0 (22.5–51.6)	29.8 (17.8–47.7)	0.215	30.9 (18.1–50.5)	31.9 (20.3–47.3)	0.818	28.1 (17.2–39.6)	36.9 (20.37–58.86)	0.034
TNFSF13B	18320.7 (7920.0–26287.8)	19822.3 (8863.2–38425.9)	0.330	22888.5 (14996.3–38425.9)	12681.6 (4363.1–30709.6)	0.025	22680.1 (12847.4–38441.8)	15814.9 (8383.3–32132.5)	0.128

On-treatment biomarker	irAE development			PD-L1 expression			CD8+ TIL density		
	Positive (N = 43)	Negative (N = 67)	*p* value	Positive (N = 63)	Negative (N = 30)	*p* value	High (N = 44)	Low (N = 45)	*p* value
CCL7	0.79 (− 8.8 –11.5)	0.0 (− 3.5 –14.5)	0.995	0.8 (− 2.2 –16.9)	0.0 (− 8.8 –3.1)	0.091	1.9 (0.0–13.8)	0.0 (− 14.5 –12.0)	0.179
CCL19	6.5 (− 8.8 –16.9)	3.5 (− 7.9 –23.5)	0.973	6.4 (− 4.2 –20.1)	3.9 (− 11.4 –19.4)	0.626	9.1 (0.0–23.6)	3.0 (− 11.4 –20.1)	0.146
CCL23	− 31.5 (− 134.4 –35.1)	13.0 (− 14.8 –88.8)	0.003	0.0 (− 95.7 –48.7)	36.8 (− 45.4 –120.3)	0.123	− 5.7 (− 87.5 –47.4)	5.3 (− 105.2 –64.2)	0.709
CCL25	3.0 (− 66.3 –59.3)	0.0 (− 47.5 –63.9)	0.910	16.2 (− 47.5 –101.0)	− 27.7 (− 66.3 –10.1)	0.007	10.3 (− 42.7 –48.7)	2.9 (− 66.3 –63.9)	0.841
IL-10	0.8 (− 11.3 –12.7)	− 1.0 (− 13.8 –14.5)	0.611	− 1.7 (− 19.8 –20.5)	0.0 (− 4.2 –1.7)	0.789	− 0.6 (− 18.3 –13.2)	− 1.0 (− 13.3 –7.3)	0.974
IL-32	0.0 (− 49.7 –32.4)	− 6.6 (− 71.6 –14.7)	0.359	0.0 (− 79.8 –113.7)	− 24.1 (− 77.1 –0.7)	0.117	− 13.5 (− 93.4 –21.3)	− 6.6 (− 51.5 –7.0)	0.879
IL-34	0.0 (− 238.7 –466.2)	0.0 (− 389.9 –11.7)	0.479	0.0 (− 483.1 –318.7)	0.0 (0.0 –0.0)	0.809	0.0 (− 606.4 –0.0)	0.0 (0.0 –516.8)	0.063
TNFSF12	− 3.5 (− 93.5 to 24.7)	− 5.4 (− 91.8 –98.8)	0.932	− 8.3 (− 96.3 –117.7)	− 3.6 (− 62.8 –13.7)	0.815	− 2.4 (− 92.7 –96.3)	− 10.6 (− 81.2 –21.8)	0.673

*CCL* chemokine ligand, *CXCL* C-X-C motif chemokine ligand, *irAE* immune related adverse event, *PD-L1* programmed cell death ligand-1, *TIL* tumor-infiltrating lymphocyte, TNFSF: tumor necrosis factor superfamily, IL: interleukin

### Correlation between pre-treatment and on-treatment biomarkers

To investigate the dynamics of soluble immune mediators in ICI treatment, the correlation between pre-treatment and on-treatment biomarker was examined. Among biomarkers of PFS, pre-treatment CXCL5 levels were significantly correlated with on-treatment changes in IL-34 (*p* = 0.039) and CCL25 (*p* = 0.019). Pre-treatment CCL17 levels were also significantly correlated with on-treatment changes in CCL25 (*p* = 0.026). For biomarkers of OS, pre-treatment CCL19 levels were significantly correlated with on-treatment changes in CCL19 (*p* = 0.002) and CCL7 (*p* = 0.009). Pre-treatment TNFSF13B levels were also significantly correlated with on-treatment change in IL-10 (*p* = 0.015). The correlation between pre-treatment and on-treatment biomarkers is shown in Supplemental Table 2A, B and Supplemental Fig. 3–6. A heatmap displaying the correlations between pre-treatment and on-treatment biomarkers is shown in Supplemental Fig. 7.

### Association of pre-treatment and on-treatment biomarkers with irAE development

The associations of pre-treatment and on-treatment biomarkers with other possible predictors were analyzed (Table 3A, B and Supplemental Fig. 8). Kaplan–Meier curves for other possible predictors are shown in Supplemental Fig. 9–12. We analyzed the association of pre-treatment and on-treatment biomarkers with the development of irAE. Of the 110 patients in this study, 43 developed one or more irAEs. The irAEs observed in this study are summarized in Supplementary Table 3. None of the pre-treatment biomarkers were significantly associated with irAE development. Among the on-treatment biomarkers, only changes in CCL23 were significantly associated with irAE development (*p* = 0.003).

**Table 3:** Correlations between (A) pre-treatment biomarkers and pre-treatment peripheral blood cells and (B) post-treatment changes in on-treatment biomarkers and post-treatment change in peripheral blood cells

Peripheral blood cells	CCL13		CCL17		CCL19		CCL21	
	r*	p**	r	*p*	r	*p*	r	*p*
WBC count	0.006	0.952	− 0.033	0.734	0.060	0.534	0.096	0.315
Neutrophil count	− 0.066	0.489	− 0.132	0.168	− 0.026	0.789	0.042	0.666
Neutrophil (%)	− 0.206	0.030	− 0.229	0.016	− 0.197	0.038	− 0.071	0.463
Lymphocyte count	0.169	0.076	0.150	0.115	0.218	0.021	0.106	0.267
Lymphocyte (%)	0.182	0.056	0.174	0.067	0.163	0.088	0.028	0.772
Monocyte count	− 0.044	0.647	0.111	0.249	0.106	0.271	0.059	0.543
Monocyte (%)	− 0.023	0.809	0.179	0.060	0.016	0.865	− 0.115	0.231
Eosinophil count	0.254	0.007	0.333	0.001	0.101	0.293	0.046	0.636
Eosinophil (%)	0.260	0.006	0.323	< 0.001	0.071	0.463	0.006	0.951
NLR	− 0.195	0.040	− 0.192	0.044	− 0.181	0.057	− 0.040	0.679

Peripheral blood cells	CXCL5		CXCL10		TNFSF13B			
	r	*p*	r	*p*	r	*p*		
WBC count	− 0.036	0.704	0.003	0.974	0.064	0.502		
Neutrophil count	− 0.103	0.281	− 0.034	0.726	0.094	0.326		
Neutrophil (%)	− 0.233	0.014	− 0.058	0.545	0.120	0.209		
Lymphocyte count	0.104	0.279	− 0.040	0.680	− 0.104	0.277		
Lymphocyte (%)	0.155	0.105	− 0.005	0.960	− 0.173	0.070		
Monocyte count	0.044	0.651	0.209	0.027	0.141	0.141		
Monocyte (%)	0.075	0.437	0.308	0.001	− 0.014	0.880		
Eosinophil count	0.114	0.236	0.018	0.852	− 0.181	0.057		
Eosinophil (%)	0.123	0.198	0.027	0.781	− 0.198	0.037		
NLR	− 0.185	0.052	− 0.013	0.891	0.162	0.089		

Peripheral blood cells	IL-10		IL-32		IL-34		CCL7	
	r *	p**	r	*p*	r	*p*	r	*p*
WBC count	− 0.414	< 0.001	− 0.192	0.044	− 0.041	0.673	0.043	0.654
Neutrophil count	− 0.376	< 0.001	− 0.153	0.109	− 0.018	0.850	0.012	0.897
Neutrophil (%)	− 0.178	0.062	− 0.064	0.505	− 0.002	0.987	− 0.062	0.519
Lymphocyte count	− 0.115	0.232	− 0.156	0.103	0.009	0.928	− 0.024	0.807
Lymphocyte (%)	0.167	0.080	0.007	0.940	0.013	0.895	− 0.014	0.883
Monocyte count	0.182	0.055	0.160	0.940	− 0.080	0.409	0.105	0.273
Monocyte (%)	0.159	0.097	0.149	0.118	− 0.084	0.385	0.179	0.060
Eosinophil count	0.010	0.913	0.028	0.769	− 0.048	0.620	0.071	0.462
Eosinophil (%)	0.080	0.403	0.070	0.469	− 0.017	0.858	0.096	0.318
NLR	− 0.171	0.073	− 0.038	0.696	0.000	0.997	− 0.053	0.579

Peripheral blood cells	CCL19		CCL23		CCL25		TNFSF12	
	r	*p*	r	*p*	r	*p*	r	*p*
WBC count	0.099	0.305	0.087	0.365	0.123	0.198	− 0.057	0.555
Neutrophil count	0.080	0.406	0.082	0.391	0.100	0.297	− 0.116	0.224
Neutrophil (%)	− 0.004	0.965	0.028	0.772	0.062	0.518	− 0.154	0.106
Lymphocyte count	− 0.043	0.658	0.059	0.538	− 0.031	0.748	0.124	0.196
Lymphocyte (%)	0.556	0.556	0.012	0.897	− 0.068	0.482	0.143	0.134
Monocyte count	− 0.008	0.937	− 0.127	0.185	− 0.012	0.897	0.106	0.270
Monocyte (%)	0.067	0.487	− 0.100	0.297	0.046	0.633	0.084	0.379
Eosinophil count	0.097	0.315	− 0.039	0.683	− 0.056	0.558	0.136	0.155
Eosinophil (%)	0.091	0.347	− 0.052	0.589	− 0.087	0.366	0.137	0.151
NLR	− 0.014	0.887	0.029	0.766	0.073	0.448	− 0.136	0.156

*CCL* chemokine ligand, *CXCL* C-X-C motif chemokine ligand, *NLR* neutrophil to lymphocyte ratio, *TNFSF* tumor necrosis factor superfamily, *WBC*, white blood cell, *IL* IL: interleukin

*r means Spearman correlation coefficient. **p means *p* value

We also analyzed the association of pre-treatment and on-treatment biomarkers with the development of Grade ≥ 3 irAE. In this study, 18 patients developed Grade ≥ 3 irAEs. There were no significant associations of any of the pre-treatment or on-treatment biomarkers with the development of Grade ≥ 3 irAEs (Supplement Table 4A and B).

### Association of pre-treatment and on-treatment biomarkers with PD-L1 expression or CD8+ TIL density

We analyzed the association of pre-treatment and on-treatment biomarkers with PD-L1 expression classified as < 1% and ≥ 1%. PD-L1 expression was evaluable in 93 patients; 30 had < 1% and 63 had ≥ 1% (24 had1-49% and 39 had ≥ 50%). Among pre-treatment biomarkers, only TNFSF13B (*p* = 0.025) was significantly associated with PD-L1 expression. In contrast, among the on-treatment biomarkers, only CCL25 was significantly associated with PD-L1 expression. Additionally, we analyzed the association of pre-treatment TNFSF13B and on-treatment changes in CCL25 with PD-L1 expression classified as < 1%, 1–49% and ≥ 50%. In this additional analysis, PD-L1 expression was not associated with pre-treatment TNFSF13B (*p* = 0.066) but was associated with on-treatment changes in CCL25 (*p* = 0.020).

CD8+ TILs density was assessed in 89 patients who were divided into high and low groups based on median values. Among pre-treatment biomarkers, CXCL10 (*p* = 0.034) was significantly associated with CD8+ TIL density. However, there was no association between on-treatment biomarkers and CD8+ TIL density.

### Correlation between pre-treatment biomarkers and pre-treatment peripheral blood cells

We analyzed the correlations between pre-treatment biomarkers and pre-treatment peripheral blood cells (Table 4A). CCL13 was significantly correlated with the proportion of neutrophils (*p* = 0.030), the eosinophil count (*p* = 0.007), the eosinophil proportion (*p* = 0.006), and NLR (*p* = 0.040). CCL17 was significantly correlated with the neutrophil proportion (*p* = 0.016), the eosinophil count (*p* = 0.001), the eosinophil proportion (*p* < 0.001) and NLR (*p* = 0.044). CCL19 was significantly correlated with the proportion of neutrophils (*p* = 0.038) and the lymphocyte count (*p* = 0.021). CXCL5 was significantly correlated with the neutrophil proportion (*p* = 0.014). CXCL10 was significantly correlated with the monocyte count (*p* = 0.027) and the monocyte proportion (*p* = 0.001). TNFSF13B was significantly correlated with the proportion of eosinophils (*p* = 0.037).

### Correlations between on-treatment biomarkers and peripheral blood cells

We also analyzed correlations between changes in on-treatment biomarkers and changes in peripheral blood cells (Table 4B). On-treatment changes in IL-10 were significantly correlated with changes in white blood cell (WBC) count (*p* < 0.001) and neutrophil count (*p* < 0.001). On-treatment changes in IL-32 were significantly correlated with changes in WBC count (*p* = 0.044). However, there were no other significant correlations between on-treatment biomarkers and peripheral blood cells.

## Discussion

In this study, we explored the association between TME and favorable outcomes of PD-1/L1 monotherapy in NSCLC. We comprehensively measured soluble immune mediators in plasma samples at ICI initiation and then 6 weeks later. Our analysis identified several potential soluble immune mediators whose pre-treatment levels or on-treatment changes were significantly associated with the prognosis in NSCLC patients treated with ICI monotherapy. Pre-treatment biomarkers were mostly chemokines such as members of the CXCL family and the CCL family, whereas on-treatment biomarkers included cytokines such as those in the IL family. Some of the levels of pre-treatment biomarkers and changes in on-treatment biomarkers were correlated, suggesting that they may reflect changes between the pre-treatment and post-treatment TME that were associated with favorable therapeutic efficacy of ICI monotherapy. To understand the complex network formation of cytokines and chemokines in the TME relevant to cancer immunotherapy, comprehensive measurement of soluble immune mediators in the same patient population as in this study was useful [1–3, 7–9]. In addition, analysis of the relationship with other potential biomarkers such as irAEs, PD-L1 expression, CD8+ TIL density and NLR is expected to provide a more detailed understanding of the TME [1–3, 16–20].

Multivariate analysis showed that pre-treatment biomarkers included 4 immune mediators, whereas on-treatment biomarkers included 5 immune mediators. These plasma biomarkers are suggested to be associated, directly or indirectly, with the cancer immunity-cycle [7–13]. CCL7, CCL23, and IL-32 attract monocytes and macrophages to regulate cancer antigen presentation [1, 7, 9, 11, 22]. CCL19, CCL21, and CCL25 promote lymphoid tissue formation, resulting in the priming and activation of effector T cell responses to cancer-specific antigens [1, 7, 12, 22]. CXCL10 traffics cytotoxic T cells to tumors [1, 7, 10, 22]. CXCL5 induces neutrophilic tumor inflammation and may negatively affect the cancer immune-cycle [7, 22]. Since it is difficult to understand the entire network of cytokines and chemokines in the cancer immune-cycle from this study alone, further large-scale studies are required.

Among the pre-treatment biomarkers, CXCL5 was associated with both PFS and OS, and the heatmap showed a survival-related trend. This suggests that CXCL5 may be an important biomarker for the therapeutic efficacy of ICI monotherapy. CXCL5, also known as neutrophil activating peptide 78, is secreted by cancer cells or other host cells in the TME, including macrophages, fibroblasts and dendritic cells [7, 23, 24]. CXCL5 recruits neutrophils into tumor tissue and promotes tumor cell proliferation and metastasis. CXCL5 was also correlated with neutrophils in peripheral blood in this study and may have promoted tumor development. CXCL5 has been reported to promote PD-L1 expression and decrease CD4+ and CD8+ TILs in tumors, but no significant association with either was observed in this study [23]. The association between CXCL5 and cancer immunotherapy is currently under investigation and has not been established [23, 24]. We found a correlation between pre-treatment CXCL5 level and on-treatment changes in IL-34 and CCL25. IL-34 modulates tumor-associated macrophage function, enhances local immune suppression, and promotes survival of cancer cells resistant to ICI treatment [9, 25, 26]. CCL25 attracts mature CD8+ T cells from the thymus into the peripheral blood [7, 12, 27, 28]. The studies on the effects of IL-34 and CCL25 on the TME may provide a better understanding of the clinical significance of CXCL5 in cancer immunotherapy.

Among the on-treatment biomarkers, changes in IL-10 and IL-32 are associated with both PFS and OS and they may reflect favorable changes in the TME that are associated with the therapeutic efficacy of ICI monotherapy. The major cellular sources of IL-10 are CD4+ T cells, CD8+ T cells, a subset of Tregs and tumor cells [8]. IL-10 is a potent suppressor of anti-tumor immunity that inhibits tumor antigen presentation [1, 8, 9]. IL-10 acts primarily on dendric cells and macrophages, and it inhibits the differentiation and antigen-presenting properties of dendric cells [1, 8]. Furthermore, in this study, on-treatment changes in IL-10 correlated with pre-treatment TNFSF13B levels, which can promote B cell activation, and on-treatment neutrophil changes in peripheral blood cells. This suggests that pre-treatment B cells and on-treatment changes of neutrophil counts may be important factors in the favorable changes of the TME after ICI administration [10, 29, 30]. IL-32 is derived from NK cells and T cells and has nine different isoforms [9, 31]. IL-32 exhibits both pro- and anti-tumor effects, but the majority of the effects promote tumor growth. IL-32 can modulate the activity of tumor-associated macrophages and induce tumor inflammation [31]. There are few reports exploring the relationship between IL-32 and cancer immunotherapy, and it is necessary to identify the function of each isoform in the cancer immune cycle [9, 31]. Cancer immunotherapy may improve patient survival by altering IL family members in the TME [1, 8, 9, 30, 31].

Since irAEs reflect immune activation by ICI administration and are associated with therapeutic efficacy, we analyzed their association with the pre- and on-treatment biomarkers identified in the present study [16, 17]. Our analysis revealed that pre-treatment biomarkers were not associated with development of irAE, whereas on-treatment changes in CCL23 were associated with the development of irAE. CCL23 is also known as CKbeta8, macrophage inflammatory protein 3 and myeloid progenitor inhibitory factor-1 [7, 11]. CCL23 is produced by eosinophils, monocytes, and monocyte-derived cells, and it acts as a chemoattractant for monocytes and dendritic cells [7, 11]. Our results support the possibility that on-treatment changes in monocytes or dendritic cells induced by CCL23 may be involved in the development of irAE associated with the therapeutic outcome of ICI monotherapy. Considering that on-treatment changes in CCL23 may play an important role in irAE development, it is expected that more detailed investigation will improve the management of irAE.

Previous studies had shown that PD-L1 could be a predictor of ICI treatment outcomes [1–6]. In this study, pre-treatment levels of TNFSF13B and on-treatment changes in CCL25 were associated with PD-L1 expression. TNFSF13B, also known as B-cell activating factor, is produced by myeloid cells, activated T cells, and bone marrow stromal cells to promote B-cell development and survival [10, 29, 30]. In solid tumors, TNFSF13B expression varies among different cancer types, and its prognostic and functional roles are not well understood [29]. An association between the presence of intra-tumoral B cells and the therapeutic efficacy of anti-PD-L1 antibodies in NSCLC has been reported, but there are no reports yet on TNFSF13B and PD-L1 expression in NSCLC specimens [30]. Since the functions of B cells in cancer immunity are not as well understood as those of T cells, further investigation is needed to determine whether TNFSF13B regulates PD-L1 expression in NSCLC. PD-L1 expression was also associated with on-treatment changes in CCL25. CCL25, also known as thymus-expressed chemokine, is expressed in the thymus, intestinal tract and tumor cells [7, 12, 27, 28]. CCL25 binds to its receptor on mature CD8+ T cells in the thymus and enhances their migration to secondary lymphoid organs such as lymph nodes. This chemoattraction of CD8+ T cells to secondary lymphoid organs may promote the therapeutic efficacy of ICI treatment [12, 27, 28]. Further studies are needed as CCL25 may be a more direct therapeutic target as a surrogate for pathological PD-L1 expression.

CD8+ TIL density is also a pathologic predictor of ICI treatment [1–3]. In this study, pre-treatment CXCL10 was associated with CD8+ TILs, but on-treatment biomarkers were not. CXCL10 showed a trend toward higher levels in the low CD8+ TIL group. CXCL10 is a chemokine that is mainly produced by intra-tumoral myeloid immune cells and it correlated with monocytes in this study. In the cancer immune cycle, CXCL10 promotes T-cell trafficking to tumors, whereas it does not promote T-cell infiltration associated with CD8+ TIL density [1, 32]. This suggests that CXCL10 in this study may have been secondarily upregulated to recruit CD8+ T cells by negative feedback, reflecting the low density of CD8 TIL+ cells. Considering that CD8+ TIL density is an important pathological predictor for cancer immunotherapy, its association with biomarkers may identify novel therapeutic targets [3].

The NLR is a predictor of ICI monotherapy that can be routinely measured in daily practice [18–20]. NLR may reflect the imbalance between pro- and anti-tumor activity of the hosts in terms of inflammatory response. Lymphocytes play an important role in tumor defense by inhibiting tumor cell proliferation and migration, whereas neutrophils have been shown to influence the cytolytic activity of lymphocytes or natural killer cells, as well as suppress T-cell proliferation [15]. In this study, pre-treatment CCL17 and CCL13 were correlated with the NLR. Although not significantly correlated with the NLR, pre-treatment CXCL5 and CCL19 were correlated with neutrophil and lymphocyte counts. These chemokines may play a role as pre-treatment biomarkers by regulating the chemoattraction of lymphocytes or neutrophils in the peripheral blood [7, 11, 12, 23, 24]. Elucidation of the role of these biomarkers in cancer immunotherapy may allow prediction of the immune status of the TME from the numbers of peripheral blood cells.

In summary, we conducted a comprehensive measurement of soluble immune mediators in plasma samples and identified several potential pre-treatment and on-treatment biomarkers in patients with NSCLC who received ICI monotherapy. It is expected that pre-treatment biomarkers might help to identify those patients who could benefit from ICI monotherapy. Moreover, on-treatment biomarkers might help our understanding of the favorable changes in TME associated with therapeutic efficacy. In addition, we found several associations and correlations between these pre- and on-treatment biomarkers and irAE, PD-L1 expression, CD8+ TIL density, and the NLR. Combining these biomarkers identified in the exploratory analysis with other predictors could provide a more detailed understanding of cancer immunotherapy and potentially identify new therapeutic targets. Nevertheless, our study had several limitations. First, this study was a retrospective single-center study. The study population was heterogeneous such as PS, driver gene mutations, and treatment lines. Second, the analysis in this study was exploratory and needs to be verified prospectively. Third, plasma samples and biopsy specimens were not collected at the same time. Forth, a validation cohort is needed because it is difficult to determine the predictive or prognostic role of soluble immune mediators from this study alone. Further large-scale studies are needed to provide a larger number of patients to better characterize the benefits of ICI monotherapy.

## Supplementary Information

Below is the link to the electronic supplementary material.

Supplementary file1 (DOCX 66 kb)

Supplementary file2 (PPTX 1597 kb)

Supplementary file3 (DOCX 12 kb)

## Data Availability

No datasets were generated or analysed during the current study.

## Abbreviations

CCL: Chemokine ligand
CTLA-4: Cytotoxic T lymphocyte antigen 4
CXCL: C-X-C motif chemokine ligand
ICI: Immune checkpoint inhibitor
IL: Interleukin
irAE: Immune-related adverse event
NLR: Neutrophil to lymphocyte ratio
NSCLC: Non-small cell lung cancer
OS: Overall survival
PD-1: Programmed cell death-1
PD-L1: Programmed cell death ligand-1
PFS: Progression-free survival
TIL: Tumor-infiltrating lymphocytes
TME: Tumor microenvironment
TNFSF: Tumor necrosis factor superfamily
WBC: White blood cell
