# Supplementary material for: Characterization of pre- and on-treatment soluble immune mediators and the tumor microenvironment in NSCLC patients receiving PD-1/L1 inhibitor monotherapy
Source: Cancer Immunol Immunother. 2024 Sep 5;73(11):214. doi: 10.1007/s00262-024-03781-8 (PMC11377373; doi:10.1007/s00262-024-03781-8)
Supplement: Supplementary file 1 — Supplementary file1 (DOCX 66 kb) [file 262_2024_3781_MOESM1_ESM.docx]

**Supplemental Table 1A. Cox proportional hazard model of associations between pre-treatment levels of soluble immune mediators and PFS, and OS**

|  | PFS | | | OS | | |
| --- | --- | --- | --- | --- | --- | --- |
| Variable | HR | 95%CI | p-value | HR | 95%CI | p-value |
| CCL1 | 0.995 | 0.973 - 1.018 | 0.686 | 0.988 | 0.965 - 1.012 | 0.325 |
| CCL2 | 1.000 | 0.996 - 1.004 | 0.892 | 1.003 | 0.999 - 1.006 | 0.206 |
| CCL3 | 1.000 | 0.996 - 1.004 | 0.860 | 1.002 | 0.998 - 1.005 | 0.341 |
| CCL7 | 0.999 | 0.994 - 1.004 | 0.799 | 1.002 | 0.997 - 1.006 | 0.551 |
| CCL8 | 1.022 | 0.977 - 1.069 | 0.337 | 1.041 | 0.989 - 1.095 | 0.127 |
| CCL11 | 1.002 | 0.993 - 1.010 | 0.722 | 1.001 | 0.992 - 1.011 | 0.801 |
| CCL13 | 1.014 | 0.995 - 1.033 | 0.149 | 1.019 | 1.000 - 1.037 | 0.045 |
| CCL15 | 1.000 | 1.000 - 1.000 | 0.759 | 1.000 | 1.000 - 1.000 | 0.952 |
| CCL17 | 1.013 | 1.000 - 1.025 | 0.043 | 1.011 | 0.997 - 1.024 | 0.126 |
| CCL19 | 1.002 | 0.998 - 1.006 | 0.319 | 1.005 | 1.001 - 1.010 | 0.022 |
| CCL20 | 0.999 | 0.951 - 1.050 | 0.970 | 0.984 | 0.933 - 1.037 | 0.550 |
| CCL21 | 1.000 | 1.000 - 1.000 | 0.434 | 1.000 | 1.000 - 1.000 | 0.050 |
| CCL22 | 1.001 | 1.000 - 1.003 | 0.094 | 1.000 | 0.999 - 1.002 | 0.518 |
| CCL23 | 0.999 | 0.998 - 1.001 | 0.216 | 1.000 | 0.998 - 1.001 | 0.548 |
| CCL24 | 1.006 | 0.996 - 1.015 | 0.256 | 1.000 | 0.992 - 1.008 | 0.996 |
| CCL25 | 1.000 | 0.999 - 1.001 | 0.572 | 1.000 | 0.999 - 1.001 | 0.523 |
| CCL26 | 0.991 | 0.971 - 1.011 | 0.363 | 0.995 | 0.975 - 1.016 | 0.652 |
| CCL27 | 1.000 | 0.999 - 1.000 | 0.285 | 1.000 | 0.999 - 1.001 | 0.891 |
| Chitinase 3-like 1 | 1.000 | 1.000 - 1.000 | 0.068 | 1.000 | 1.000 - 1.000 | 0.670 |
| CX3CL1 | 1.000 | 0.998 - 1.002 | 0.757 | 1.000 | 0.998 - 1.003 | 0.718 |
| CXCL1 | 1.001 | 0.997 - 1.004 | 0.775 | 1.001 | 0.997 - 1.004 | 0.739 |
| CXCL2 | 1.000 | 0.992 - 1.007 | 0.959 | 1.005 | 0.997 - 1.013 | 0.224 |
| CXCL5 | 1.001 | 1.000 - 1.002 | 0.006 | 1.001 | 1.000 - 1.002 | 0.006 |
| CXCL6 | 0.985 | 0.944 - 1.028 | 0.495 | 0.982 | 0.937 - 1.028 | 0.435 |
| CXCL8 | 1.001 | 1.000 - 1.002 | 0.117 | 1.000 | 1.000 - 1.001 | 0.316 |
| CXCL9 | 1.000 | 0.999 - 1.001 | 0.714 | 1.000 | 0.999 - 1.001 | 0.996 |
| CXCL10 | 1.004 | 1.001 - 1.007 | 0.009 | 1.002 | 0.999 - 1.005 | 0.122 |
| CXCL11 | 1.014 | 0.985 - 1.043 | 0.343 | 1.007 | 0.978 - 1.036 | 0.658 |
| CXCL12 | 1.000 | 1.000 - 1.001 | 0.190 | 1.000 | 1.000 - 1.001 | 0.312 |
| CXCL13 | 1.017 | 0.984 - 1.050 | 0.325 | 1.026 | 0.991 - 1.062 | 0.146 |
| CXCL16 | 1.000 | 0.999 - 1.001 | 0.547 | 1.000 | 0.999 - 1.001 | 0.761 |
| GM-CSF | 0.972 | 0.941 - 1.004 | 0.086 | 0.975 | 0.943 - 1.008 | 0.142 |
| IFN-a2 | 1.000 | 1.000 - 1.000 | 0.782 | 1.000 | 1.000 - 1.000 | 0.824 |
| IFN-b | 1.001 | 0.998 - 1.003 | 0.593 | 1.000 | 0.997 - 1.002 | 0.688 |
| IFN-g | 1.001 | 0.998 - 1.003 | 0.574 | 1.000 | 0.998 - 1.002 | 0.957 |
| IL-1b | 0.965 | 0.848 - 1.098 | 0.589 | 1.007 | 0.884 - 1.147 | 0.921 |
| IL-2 | 1.000 | 0.999 - 1.001 | 0.728 | 1.000 | 0.999 - 1.001 | 0.934 |
| IL-4 | 0.996 | 0.984 - 1.009 | 0.539 | 1.001 | 0.989 - 1.014 | 0.842 |
| IL-6 | 0.998 | 0.988 - 1.008 | 0.686 | 1.001 | 0.992 - 1.011 | 0.789 |
| IL-10 | 1.000 | 0.997 - 1.003 | 0.968 | 1.000 | 0.997 - 1.003 | 0.797 |
| IL-11 | 1.002 | 0.996 - 1.008 | 0.604 | 1.001 | 0.996 - 1.006 | 0.615 |
| IL-12(p40) | 1.000 | 1.000 - 1.000 | 0.659 | 1.000 | 1.000 - 1.000 | 0.866 |
| IL-12(p70) | 1.000 | 0.999 - 1.002 | 0.637 | 1.000 | 0.998 - 1.002 | 0.965 |
| IL-16 | 1.000 | 0.998 - 1.002 | 0.904 | 0.999 | 0.997 - 1.001 | 0.461 |
| IL-19 | 0.999 | 0.997 - 1.002 | 0.677 | 1.000 | 0.998 - 1.003 | 0.880 |
| IL-20 | 1.000 | 0.998 - 1.003 | 0.830 | 0.999 | 0.997 - 1.002 | 0.557 |
| IL-22 | 1.000 | 1.000 - 1.000 | 0.947 | 1.000 | 1.000 - 1.000 | 0.980 |
| IL-26 | 1.000 | 1.000 - 1.000 | 0.708 | 1.000 | 1.000 - 1.000 | 0.654 |
| IL-27(p28) | 1.000 | 0.999 - 1.001 | 0.764 | 1.000 | 0.999 - 1.001 | 0.719 |
| IL-29 | 1.000 | 1.000 - 1.000 | 0.653 | 1.000 | 1.000 - 1.000 | 0.949 |
| IL-32 | 1.000 | 1.000 - 1.001 | 0.579 | 1.000 | 1.000 - 1.000 | 0.840 |
| IL-34 | 1.000 | 1.000 - 1.000 | 0.146 | 1.000 | 1.000 - 1.000 | 0.417 |
| IL-35 | 1.000 | 1.000 - 1.000 | 0.628 | 1.000 | 1.000 - 1.000 | 0.966 |
| IL-6Ra | 1.000 | 1.000 - 1.000 | 0.755 | 1.000 | 1.000 - 1.000 | 0.895 |
| MIF | 1.000 | 1.000 - 1.000 | 0.167 | 1.000 | 1.000 - 1.000 | 0.463 |
| MMP-1 | 1.000 | 1.000 - 1.000 | 0.685 | 1.000 | 1.000 - 1.000 | 0.690 |
| MMP-2 | 1.000 | 1.000 - 1.000 | 0.779 | 1.000 | 1.000 - 1.000 | 0.400 |
| MMP-3 | 1.000 | 1.000 - 1.000 | 0.626 | 1.000 | 1.000 - 1.000 | 0.699 |
| Osteocalcin | 1.000 | 1.000 - 1.000 | 0.482 | 1.000 | 1.000 - 1.000 | 0.335 |
| Osteopontin | 1.000 | 1.000 - 1.000 | 0.339 | 1.000 | 1.000 - 1.000 | 0.131 |
| Pentraxin-3 | 1.000 | 1.000 - 1.000 | 0.454 | 1.000 | 1.000 - 1.000 | 0.056 |
| TNF-a | 0.999 | 0.988 - 1.009 | 0.785 | 1.003 | 0.993 - 1.014 | 0.544 |
| sCD163 | 1.000 | 1.000 - 1.000 | 0.205 | 1.000 | 1.000 - 1.000 | 0.349 |
| sIL-6Rb | 1.000 | 1.000 - 1.000 | 0.879 | 1.000 | 1.000 - 1.000 | 0.696 |
| sTNF-R1 | 1.000 | 1.000 - 1.000 | 0.906 | 1.000 | 1.000 - 1.000 | 0.495 |
| sTNF-R2 | 1.000 | 1.000 - 1.000 | 0.420 | 1.000 | 1.000 - 1.000 | 0.329 |
| TNFRSF8 | 1.000 | 1.000 - 1.000 | 0.646 | 1.000 | 1.000 - 1.000 | 0.837 |
| TNFSF12 | 1.000 | 0.999 - 1.001 | 0.839 | 1.000 | 0.999 - 1.001 | 0.856 |
| TNFSF13 | 1.000 | 1.000 - 1.000 | 0.813 | 1.000 | 1.000 - 1.000 | 0.962 |
| TNFSF13B | 1.000 | 1.000 - 1.000 | 0.055 | 1.000 | 1.000 - 1.000 | 0.028 |
| TNFSF14 | 1.000 | 1.000 - 1.000 | 0.588 | 1.000 | 1.000 - 1.000 | 0.695 |
| TSLP | 1.001 | 0.998 - 1.003 | 0.589 | 1.000 | 0.998 - 1.002 | 0.998 |
| VEGF | 0.999 | 0.990 - 1.009 | 0.915 | 1.000 | 0.991 - 1.010 | 0.973 |

CCL: chemokine ligands, CI: confidence interval, CXCL: C-X-C motif chemokine ligands, GM-CSF: granulocyte macrophage colony-stimulating factor, HR: hazard ratio, IL: interleukin, IFN: interferon, MIF: macrophage migration inhibitory factor, MMP: matrix metalloproteinase, OS: overall survival, PFS: progression-free survival, TNF: tumor necrosis factor, TNFRSF: tumor necrosis factor receptor superfamily, TNFSF: tumor necrosis factor superfamily, TSLP: thymic stromal lymphopoietin, VEGF: vascular endothelial growth factor.

**Supplemental Table 1B. Cox proportional hazard model of associations between on-treatment changes in soluble immune mediators and PFS, and OS**

|  | PFS | | | OS | | |
| --- | --- | --- | --- | --- | --- | --- |
| Variables | HR | 95%CI | p-value | HR | 95%CI | p-value |
| CCL1 | 0.984 | 0.964 - 1.004 | 0.123 | 0.985 | 0.960 - 1.010 | 0.223 |
| CCL2 | 1.000 | 0.997 - 1.002 | 0.727 | 1.000 | 0.997 - 1.003 | 0.836 |
| CCL3 | 1.001 | 0.998 - 1.003 | 0.684 | 1.000 | 0.997 - 1.003 | 0.923 |
| CCL7 | 0.995 | 0.989 - 1.000 | 0.066 | 0.993 | 0.988 - 0.999 | 0.023 |
| CCL8 | 0.970 | 0.933 - 1.008 | 0.115 | 0.976 | 0.932 - 1.022 | 0.297 |
| CCL11 | 0.994 | 0.982 - 1.006 | 0.332 | 0.995 | 0.982 - 1.008 | 0.460 |
| CCL13 | 0.996 | 0.983 - 1.010 | 0.577 | 0.998 | 0.983 - 1.012 | 0.741 |
| CCL15 | 1.000 | 1.000 - 1.000 | 0.632 | 1.000 | 1.000 - 1.000 | 0.606 |
| CCL17 | 0.992 | 0.981 - 1.003 | 0.166 | 0.990 | 0.980 - 1.001 | 0.079 |
| CCL19 | 0.998 | 0.994 - 1.002 | 0.312 | 0.995 | 0.990 - 0.999 | 0.019 |
| CCL20 | 0.998 | 0.956 - 1.043 | 0.936 | 1.000 | 0.959 - 1.042 | 0.982 |
| CCL21 | 1.000 | 1.000 - 1.000 | 0.052 | 1.000 | 1.000 - 1.000 | 0.056 |
| CCL22 | 0.999 | 0.997 - 1.001 | 0.314 | 1.000 | 0.998 - 1.001 | 0.758 |
| CCL23 | 1.001 | 1.000 - 1.002 | 0.042 | 1.001 | 1.000 - 1.002 | 0.124 |
| CCL24 | 0.996 | 0.987 - 1.005 | 0.352 | 0.997 | 0.988 - 1.006 | 0.474 |
| CCL25 | 0.999 | 0.998 - 1.000 | 0.014 | 0.999 | 0.998 - 1.000 | 0.054 |
| CCL26 | 0.983 | 0.958 - 1.010 | 0.214 | 0.982 | 0.956 - 1.010 | 0.205 |
| CCL27 | 1.000 | 1.000 - 1.001 | 0.604 | 1.000 | 1.000 - 1.001 | 0.655 |
| Chitinase 3-like 1 | 1.000 | 1.000 - 1.000 | 0.979 | 1.000 | 1.000 - 1.000 | 0.611 |
| CX3CL1 | 1.001 | 0.999 - 1.002 | 0.497 | 1.000 | 0.998 - 1.001 | 0.821 |
| CXCL1 | 1.000 | 0.999 - 1.001 | 0.548 | 1.000 | 0.999 - 1.001 | 0.834 |
| CXCL2 | 0.997 | 0.991 - 1.004 | 0.407 | 0.994 | 0.987 - 1.001 | 0.084 |
| CXCL5 | 1.000 | 0.999 - 1.001 | 0.883 | 1.000 | 0.999 - 1.001 | 0.897 |
| CXCL6 | 0.970 | 0.930 - 1.012 | 0.162 | 0.961 | 0.922 - 1.002 | 0.065 |
| CXCL8 | 1.000 | 1.000 - 1.001 | 0.844 | 1.000 | 1.000 - 1.001 | 0.534 |
| CXCL9 | 1.000 | 1.000 - 1.001 | 0.227 | 1.000 | 1.000 - 1.001 | 0.372 |
| CXCL10 | 0.999 | 0.997 - 1.002 | 0.499 | 1.000 | 0.998 - 1.002 | 0.993 |
| CXCL11 | 0.998 | 0.976 - 1.020 | 0.825 | 1.004 | 0.982 - 1.027 | 0.714 |
| CXCL12 | 1.000 | 0.999 - 1.000 | 0.125 | 1.000 | 0.999 - 1.000 | 0.455 |
| CXCL13 | 1.010 | 0.995 - 1.026 | 0.178 | 1.010 | 0.993 - 1.026 | 0.260 |
| CXCL16 | 1.000 | 0.999 - 1.002 | 0.484 | 1.000 | 0.999 - 1.002 | 0.424 |
| GM-CSF | 0.998 | 0.967 - 1.030 | 0.899 | 1.003 | 0.970 - 1.037 | 0.852 |
| IFN-a2 | 1.000 | 1.000 - 1.000 | 0.167 | 1.000 | 0.999 - 1.000 | 0.126 |
| IFN-b | 1.001 | 0.998 - 1.004 | 0.599 | 1.001 | 0.998 - 1.004 | 0.653 |
| IFN-g | 0.993 | 0.980 - 1.006 | 0.273 | 0.992 | 0.980 - 1.004 | 0.202 |
| IL-1b | 1.008 | 0.872 - 1.166 | 0.909 | 0.994 | 0.855 - 1.156 | 0.940 |
| IL-2 | 0.999 | 0.998 - 1.000 | 0.163 | 0.999 | 0.998 - 1.000 | 0.146 |
| IL-4 | 0.985 | 0.966 - 1.003 | 0.100 | 0.985 | 0.964 - 1.006 | 0.155 |
| IL-6 | 1.004 | 0.996 - 1.012 | 0.306 | 1.001 | 0.993 - 1.009 | 0.843 |
| IL-10 | 0.998 | 0.997 - 1.000 | 0.041 | 0.998 | 0.996 - 1.000 | 0.037 |
| IL-11 | 0.997 | 0.993 - 1.002 | 0.257 | 0.996 | 0.990 - 1.001 | 0.136 |
| IL-12(p40) | 1.000 | 1.000 - 1.000 | 0.848 | 1.000 | 1.000 - 1.000 | 0.756 |
| IL-12(p70) | 0.997 | 0.993 - 1.001 | 0.196 | 0.997 | 0.993 - 1.001 | 0.178 |
| IL-16 | 1.000 | 0.998 - 1.001 | 0.792 | 1.000 | 0.998 - 1.001 | 0.735 |
| IL-19 | 1.000 | 0.998 - 1.001 | 0.795 | 1.000 | 0.998 - 1.001 | 0.730 |
| IL-20 | 0.999 | 0.996 - 1.002 | 0.501 | 0.999 | 0.996 - 1.001 | 0.242 |
| IL-22 | 1.000 | 1.000 - 1.000 | 0.081 | 1.000 | 1.000 - 1.000 | 0.056 |
| IL-26 | 1.000 | 1.000 - 1.000 | 0.256 | 1.000 | 1.000 - 1.000 | 0.244 |
| IL-27(p28) | 1.000 | 0.999 - 1.000 | 0.390 | 1.000 | 0.999 - 1.000 | 0.443 |
| IL-29 | 0.999 | 0.999 - 1.000 | 0.065 | 0.999 | 0.999 - 1.000 | 0.078 |
| IL-32 | 0.998 | 0.997 - 0.999 | 0.004 | 0.998 | 0.997 - 0.999 | 0.005 |
| IL-34 | 1.000 | 1.000 - 1.000 | 0.039 | 1.000 | 1.000 - 1.000 | 0.056 |
| IL-35 | 1.000 | 1.000 - 1.000 | 0.530 | 1.000 | 0.999 - 1.000 | 0.327 |
| IL-6Ra | 1.000 | 1.000 - 1.000 | 0.698 | 1.000 | 1.000 - 1.000 | 0.826 |
| MIF | 1.000 | 1.000 - 1.000 | 0.063 | 1.000 | 1.000 - 1.000 | 0.270 |
| MMP-1 | 1.000 | 1.000 - 1.000 | 0.723 | 1.000 | 1.000 - 1.000 | 0.793 |
| MMP-2 | 1.000 | 1.000 - 1.000 | 0.220 | 1.000 | 1.000 - 1.000 | 0.067 |
| MMP-3 | 1.000 | 1.000 - 1.000 | 0.339 | 1.000 | 1.000 - 1.000 | 0.783 |
| Osteocalcin | 1.000 | 1.000 - 1.000 | 0.342 | 1.000 | 1.000 - 1.000 | 0.749 |
| Osteopontin | 1.000 | 1.000 - 1.000 | 0.782 | 1.000 | 1.000 - 1.000 | 0.543 |
| Pentraxin-3 | 1.000 | 1.000 - 1.000 | 0.823 | 1.000 | 1.000 - 1.000 | 0.606 |
| TNF-a | 0.992 | 0.981 - 1.004 | 0.217 | 0.993 | 0.976 - 1.011 | 0.464 |
| sCD163 | 1.000 | 1.000 - 1.000 | 0.230 | 1.000 | 1.000 - 1.000 | 0.630 |
| sIL-6Rb | 1.000 | 1.000 - 1.000 | 0.134 | 1.000 | 1.000 - 1.000 | 0.187 |
| sTNF-R1 | 1.000 | 1.000 - 1.000 | 0.648 | 1.000 | 1.000 - 1.000 | 0.465 |
| sTNF-R2 | 1.000 | 1.000 - 1.000 | 0.076 | 1.000 | 1.000 - 1.000 | 0.104 |
| TNFRSF8 | 1.000 | 1.000 - 1.000 | 0.576 | 1.000 | 1.000 - 1.000 | 0.596 |
| TNFSF12 | 0.999 | 0.998 - 1.000 | 0.009 | 0.999 | 0.998 - 1.000 | 0.077 |
| TNFSF13 | 1.000 | 1.000 - 1.000 | 0.194 | 1.000 | 1.000 - 1.000 | 0.218 |
| TNFSF13B | 1.000 | 1.000 - 1.000 | 0.553 | 1.000 | 1.000 - 1.000 | 0.496 |
| TNFSF14 | 1.000 | 1.000 - 1.000 | 0.087 | 1.000 | 1.000 - 1.000 | 0.066 |
| TSLP | 0.997 | 0.994 - 1.000 | 0.087 | 0.997 | 0.994 - 1.001 | 0.127 |
| VEGF | 1.002 | 0.998 - 1.006 | 0.405 | 1.002 | 0.997 - 1.007 | 0.417 |

CCL: chemokine ligands, CI: confidence interval, CXCL: C-X-C motif chemokine ligands, GM-CSF: granulocyte macrophage colony-stimulating factor, HR: hazard ratio, IL: interleukin, IFN: interferon, MIF: macrophage migration inhibitory factor, MMP: matrix metalloproteinase, OS: overall survival, PFS: progression-free survival, TNF: tumor necrosis factor, TNFRSF: tumor necrosis factor receptor superfamily, TNFSF: tumor necrosis factor superfamily, TSLP: thymic stromal lymphopoietin, VEGF: vascular endothelial growth factor.

**Supplemental Table 2A. Correlation between the levels of pre-treatment biomarkers and changes in on-treatment biomarkers for progression-free survival**

|  | On-treatment biomarker | | | | | |
| --- | --- | --- | --- | --- | --- | --- |
| Pre-treatment  Biomarker | CCL23 | | CCL25 | | IL-10 | |
|  | Spearman | p-value | Spearman | p-value | Spearman | p-value |
| CCL17 | 0.025 | 0.792 | -0.212 | 0.026 | -0.118 | 0.220 |
| CXCL5 | 0.009 | 0.928 | -0.223 | 0.019 | -0.074 | 0.446 |
| CXCL10 | -0.105 | 0.274 | -0.040 | 0.676 | -0.155 | 0.106 |
|  | On-treatment biomarker | | | | | |
| Pre-treatment  Biomarker | IL-32 | | IL-34 | | TNFSF12 | |
|  | Spearman | p-value | Spearman | p-value | Spearman | p-value |
| CCL17 | -0.105 | 0.277 | -0.104 | 0.283 | -0.139 | 0.147 |
| CXCL5 | -0.055 | 0.566 | -0.198 | 0.039 | -0.020 | 0.832 |
| CXCL10 | -0.040 | 0.676 | -0.048 | 0.620 | 0.031 | 0.750 |

CCL: chemokine ligands, CXCL: C-X-C motif chemokine ligands, IL: interleukin, TNFSF: tumor necrosis factor super family.

**Supplemental Table 2B. Correlation between the levels of pre-treatment biomarkers and changes in on-treatment biomarkers for overall survival**

|  | On-treatment biomarker | | | |
| --- | --- | --- | --- | --- |
| Pre-treatment  Biomarker | CCL7 | | CCL19 | |
|  | Spearman | p-value | Spearman | p-value |
| CCL13 | -0.147 | 0.124 | -0.165 | 0.087 |
| CCL19 | -0.248 | 0.009 | -0.301 | 0.002 |
| CCL21 | -0.131 | 0.173 | -0.173 | 0.072 |
| CXCL5 | -0.060 | 0.531 | 0.046 | 0.632 |
| TNFSF13B | -0.050 | 0.605 | -0.089 | 0.358 |
|  | On-treatment biomarker | | | |
| Pre-treatment  Biomarker | IL-10 | | IL-32 | |
|  | Spearman | p-value | Spearman | p-value |
| CCL13 | 0.060 | 0.531 | 0.058 | 0.550 |
| CCL19 | -0.112 | 0.246 | -0.121 | 0.209 |
| CCL21 | -0.130 | 0.178 | -0.140 | 0.143 |
| CXCL5 | -0.074 | 0.446 | -0.055 | 0.566 |
| TNFSF13B | -0.231 | 0.015 | -0.144 | 0.134 |

CCL: chemokine ligands, CXCL: C-X-C motif chemokine ligands, IL: interleukin, TNFSF: tumor necrosis factor super family.

**Supplemental Table 3. Summary of irAE**

| Type of irAE | All Grades | Grade ≥ 3 |
| --- | --- | --- |
| Dermatitis | 16 | 1 |
| Pneumonitis | 15 | 10 |
| Hepatitis | 10 | 2 |
| Fatigue | 7 | 0 |
| Thyroiditis | 6 | 2 |
| Colitis | 5 | 2 |
| Arthritis | 5 | 0 |
| Anorexia | 3 | 0 |
| Fever | 3 | 0 |
| Other | 4 | 1 |

irAE: immune related adverse event

**Supplemental Table 4A. Association between the levels of pre-treatment biomarkers and development of Grade 3 ≥ irAEs**

| Pre-treatment  Variable | irAE (-)  (N=92) | irAE (+)  (N=18) | p-value |
| --- | --- | --- | --- |
| CCL13 | 18.5  [14.6 – 25.7] | 18.9  [15.1 – 23.4] | 0.987 |
| CCL17 | 9.1  [4.1 – 17.2] | 8.3  [3.3 – 22.5] | 0.984 |
| CCL19 | 35.9  [20.3 – 53.8] | 43.4  [22.7 – 52.0] | 0.605 |
| CCL21 | 6554.6  [1801.0 – 8697.8] | 6655.6  [4513.0 – 8497.4] | 0.654 |
| CXLC5 | 5.7  [5.7 – 69.5] | 5.7  [5.7 – 124.4] | 0.845 |
| CXCL10 | 29.8  [19.7 – 47.2] | 36.5  [29.8 – 58.7] | 0.068 |
| TNFSF13B | 19488.2  [8854.9 – 35128.7] | 19398.5  [9508.4 – 24528.6] | 0.824 |

CCL: chemokine ligands, CXCL: C-X-C motif chemokine ligands, irAE: immune related adverse event, TNFSF: tumor necrosis factor super family.

**Supplemental Table 4B. Association between changes in on-treatment biomarkers and development of Grade 3 ≥ irAEs**

| Post-treatment  Change | irAE (-)  (N=92) | irAE (+)  (N=18) | p-value |
| --- | --- | --- | --- |
| CCL7 | 0.0  [-3.6 – 11.3] | 5.2  [-5.5 – 12.2] | 0.41 |
| CCL19 | 4.1  [-9.4 – 18.4] | 9.1  [0.5 – 23.5] | 0.48 |
| CCL23 | 4.7  [-77.7 – 72.0] | -10.0  [-106.5 – 49.6] | 0.44 |
| CCL25 | 3.0  [-51.8 – 63.8] | -0.8  [-61.5 – 53.3] | 0.72 |
| IL-10 | 0.1  [-8.8 – 13.5] | -4.6  [-25.5 – 0.2] | 0.12 |
| IL-32 | 0.0  [-47.2 – 27.0] | -15.1  [-88.6 – 1.7] | 0.35 |
| IL-34 | 0.0  [-238.7 – 156.9] | 0.0  [-606.4 – 238.7] | 0.94 |
| TNFSF12 | -3.3  [-92.7 – 31.2] | -4.1  [-88.2 – 42.5] | 0.93 |

CCL: chemokine ligands, IL: interleukin, irAE: immune related adverse event, TNFSF: tumor necrosis factor super family.
