# Supplementary material for: Characterization of pre- and on-treatment soluble immune mediators and the tumor microenvironment in NSCLC patients receiving PD-1/L1 inhibitor monotherapy
Source: Cancer Immunol Immunother. 2024 Sep 5;73(11):214. doi: 10.1007/s00262-024-03781-8 (PMC11377373; doi:10.1007/s00262-024-03781-8)
Supplement: Supplementary file 2 — Supplementary file2 (PPTX 1597 kb) [file 262_2024_3781_MOESM2_ESM.pptx]

## Slide 1
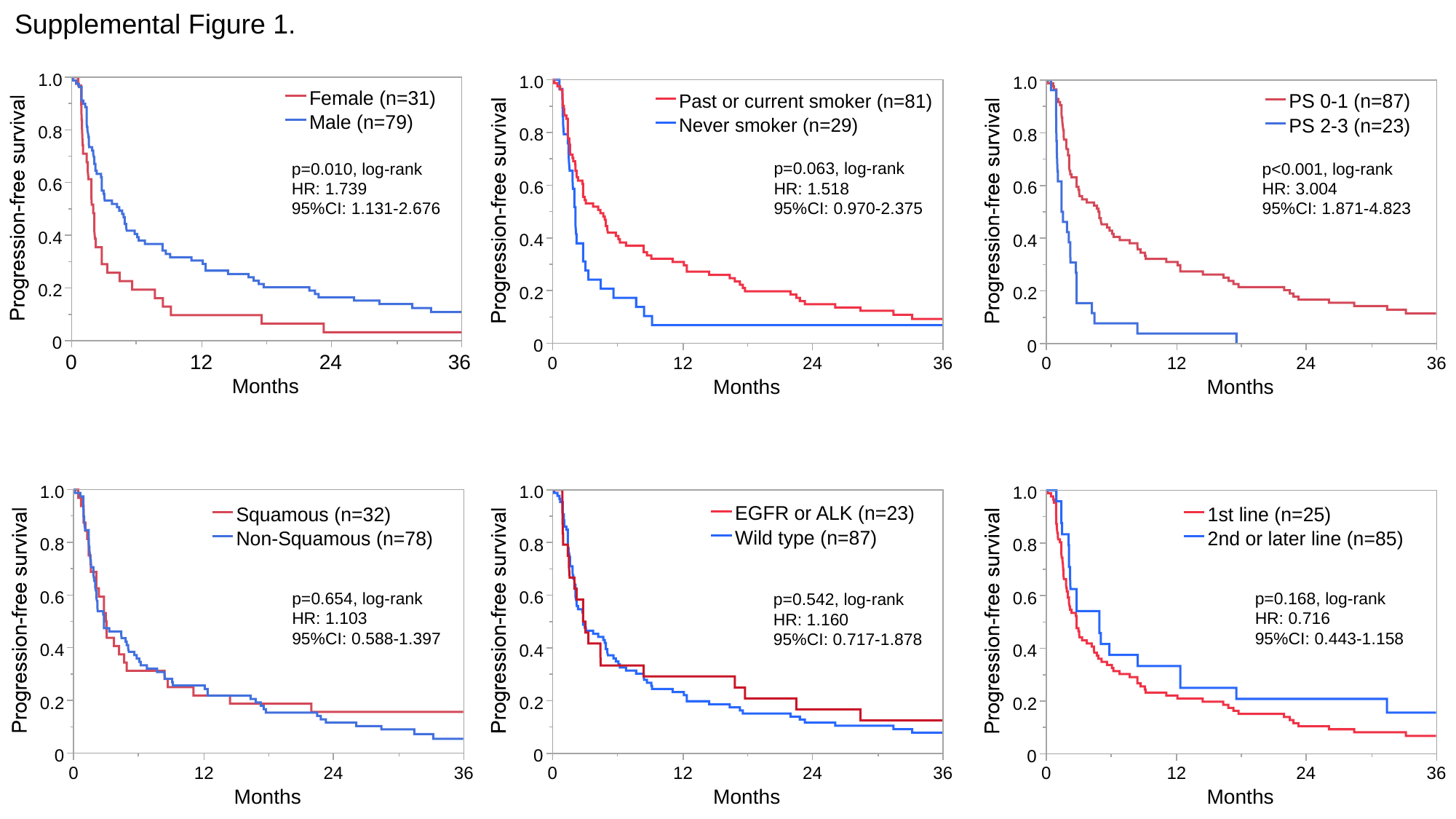

Supplemental Figure 1.
p=0.063, log-rank
HR: 1.518
95%CI: 0.970-2.375
p=0.010, log-rank
HR: 1.739
95%CI: 1.131-2.676
p<0.001, log-rank
HR: 3.004
95%CI: 1.871-4.823
p=0.168, log-rank
HR: 0.716
95%CI: 0.443-1.158
p=0.654, log-rank
HR: 1.103
95%CI: 0.588-1.397
p=0.542, log-rank
HR: 1.160
95%CI: 0.717-1.878

## Slide 2
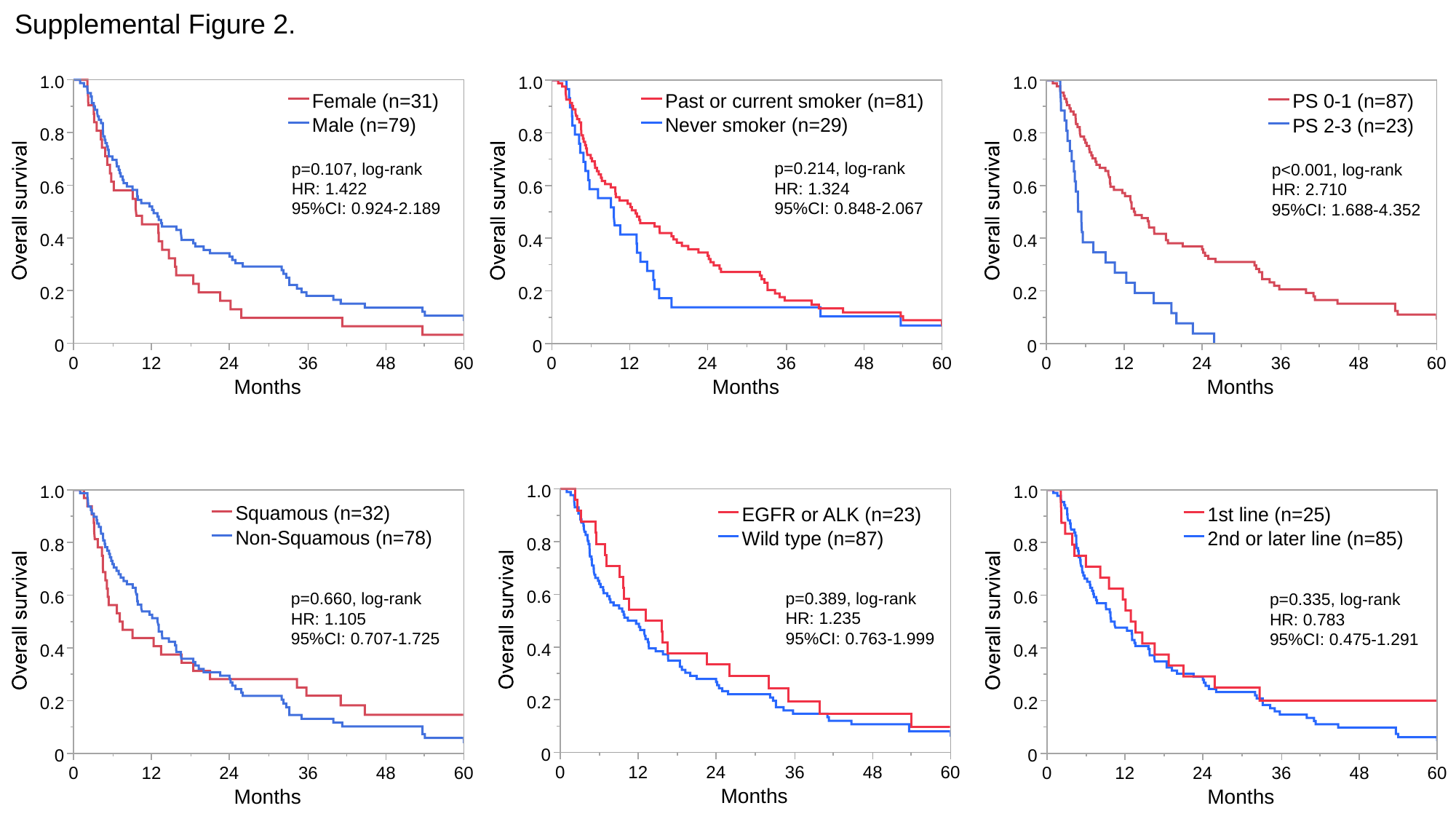

Supplemental Figure 2.
p=0.214, log-rank
HR: 1.324
95%CI: 0.848-2.067
p=0.107, log-rank
HR: 1.422
95%CI: 0.924-2.189
p<0.001, log-rank
HR: 2.710
95%CI: 1.688-4.352
p=0.389, log-rank
HR: 1.235
95%CI: 0.763-1.999
p=0.660, log-rank
HR: 1.105
95%CI: 0.707-1.725
p=0.335, log-rank
HR: 0.783
95%CI: 0.475-1.291

## Slide 3
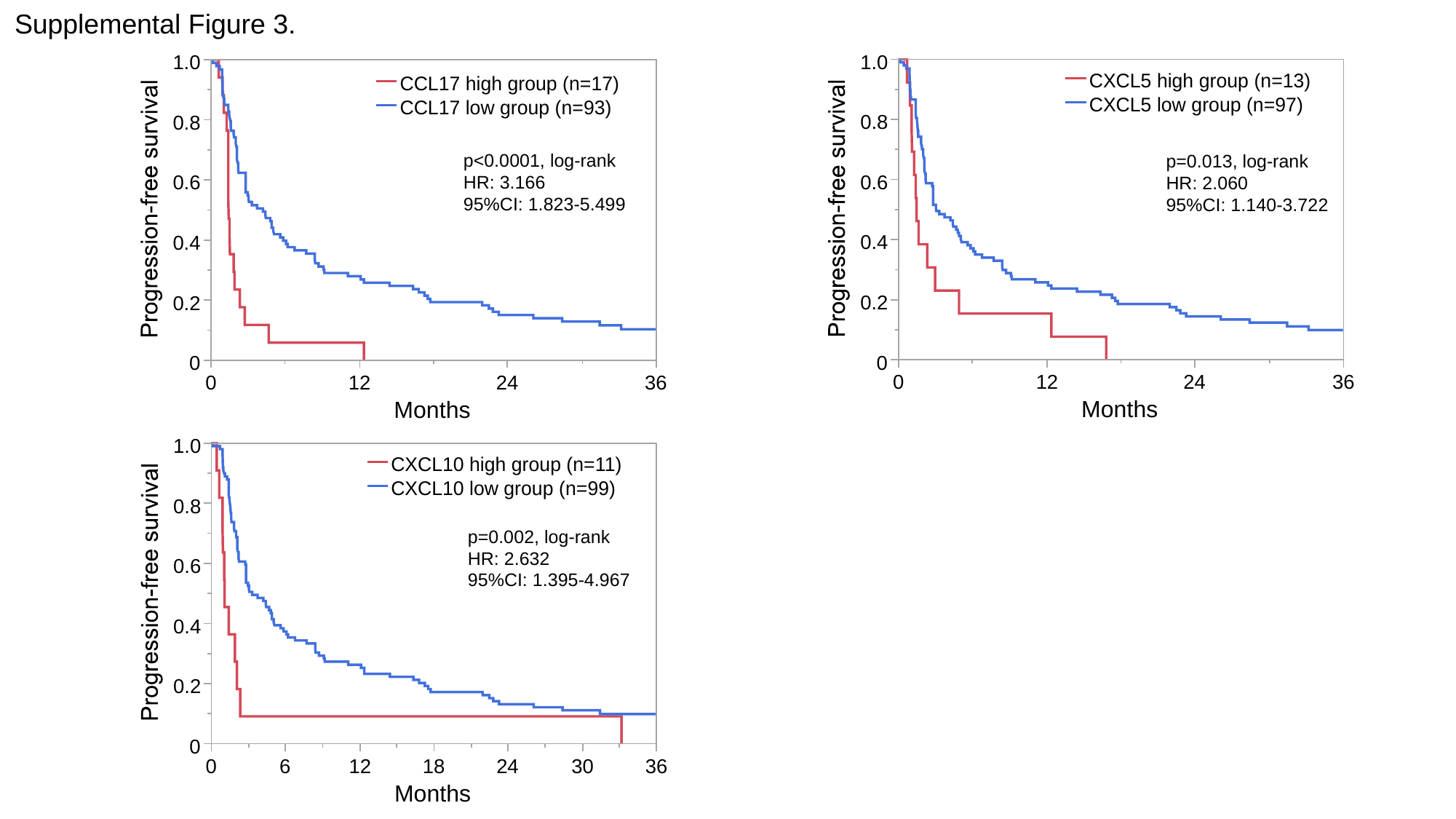

Supplemental Figure 3.
p<0.0001, log-rank
HR: 3.166
95%CI: 1.823-5.499
p=0.013, log-rank
HR: 2.060
95%CI: 1.140-3.722
p=0.002, log-rank
HR: 2.632
95%CI: 1.395-4.967

## Slide 4
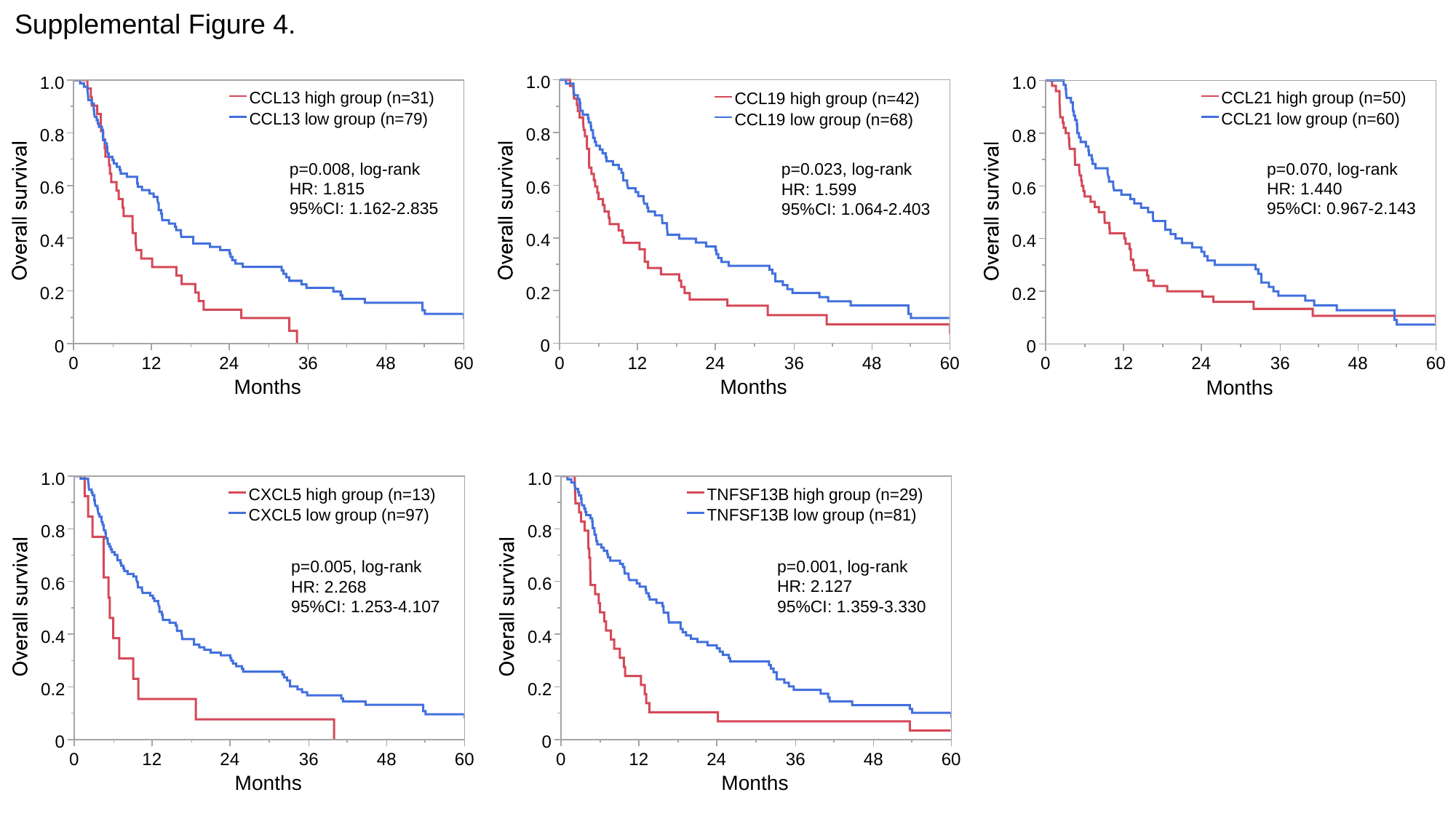

Supplemental Figure 4.
p=0.070, log-rank
HR: 1.440
95%CI: 0.967-2.143
p=0.008, log-rank
HR: 1.815
95%CI: 1.162-2.835
p=0.023, log-rank
HR: 1.599
95%CI: 1.064-2.403
p=0.001, log-rank
HR: 2.127
95%CI: 1.359-3.330
p=0.005, log-rank
HR: 2.268
95%CI: 1.253-4.107

## Slide 5
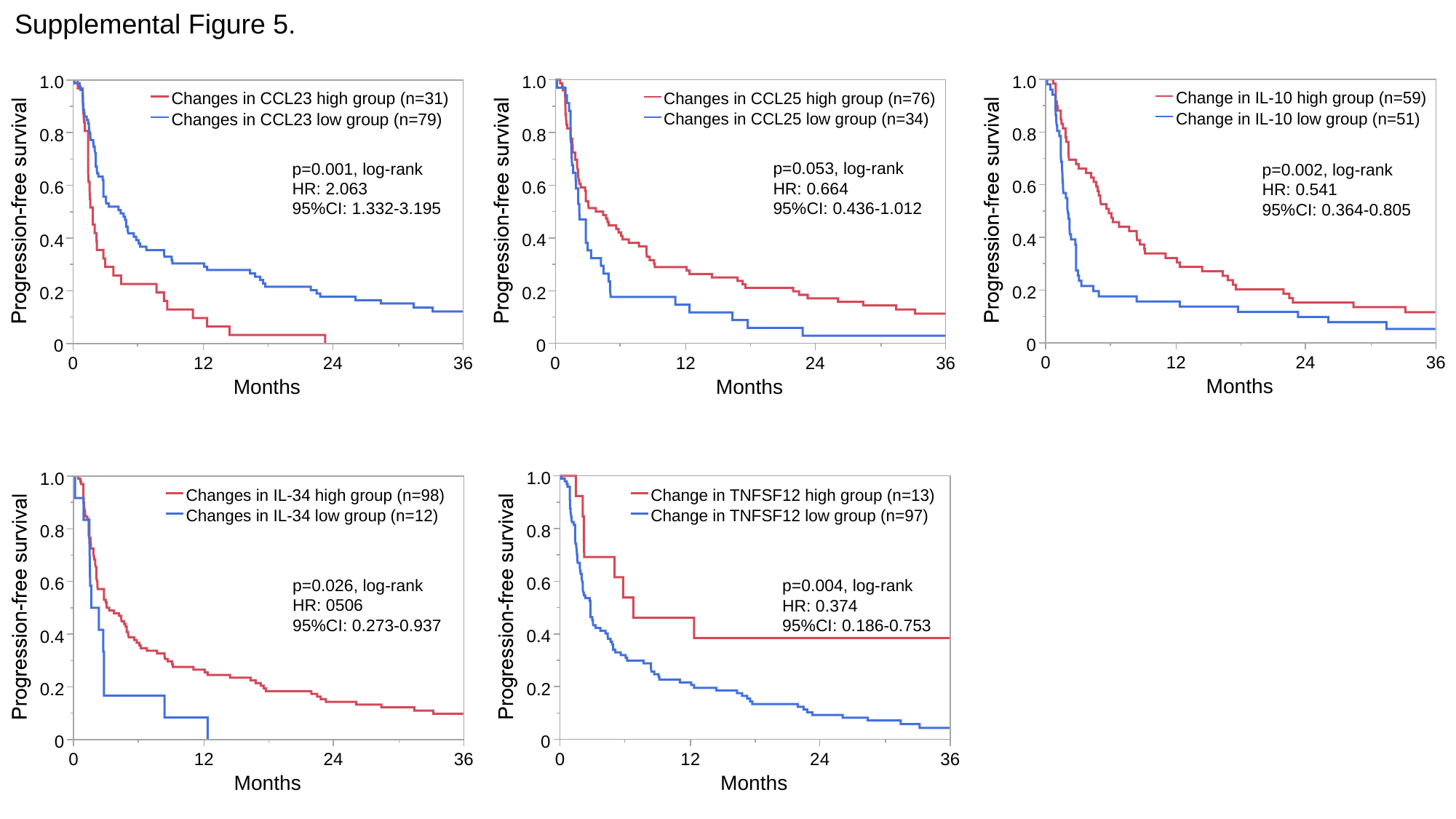

Supplemental Figure 5.
p=0.053, log-rank
HR: 0.664
95%CI: 0.436-1.012
p=0.001, log-rank
HR: 2.063
95%CI: 1.332-3.195
p=0.002, log-rank
HR: 0.541
95%CI: 0.364-0.805
p=0.026, log-rank
HR: 0506
95%CI: 0.273-0.937
p=0.004, log-rank
HR: 0.374
95%CI: 0.186-0.753

## Slide 6
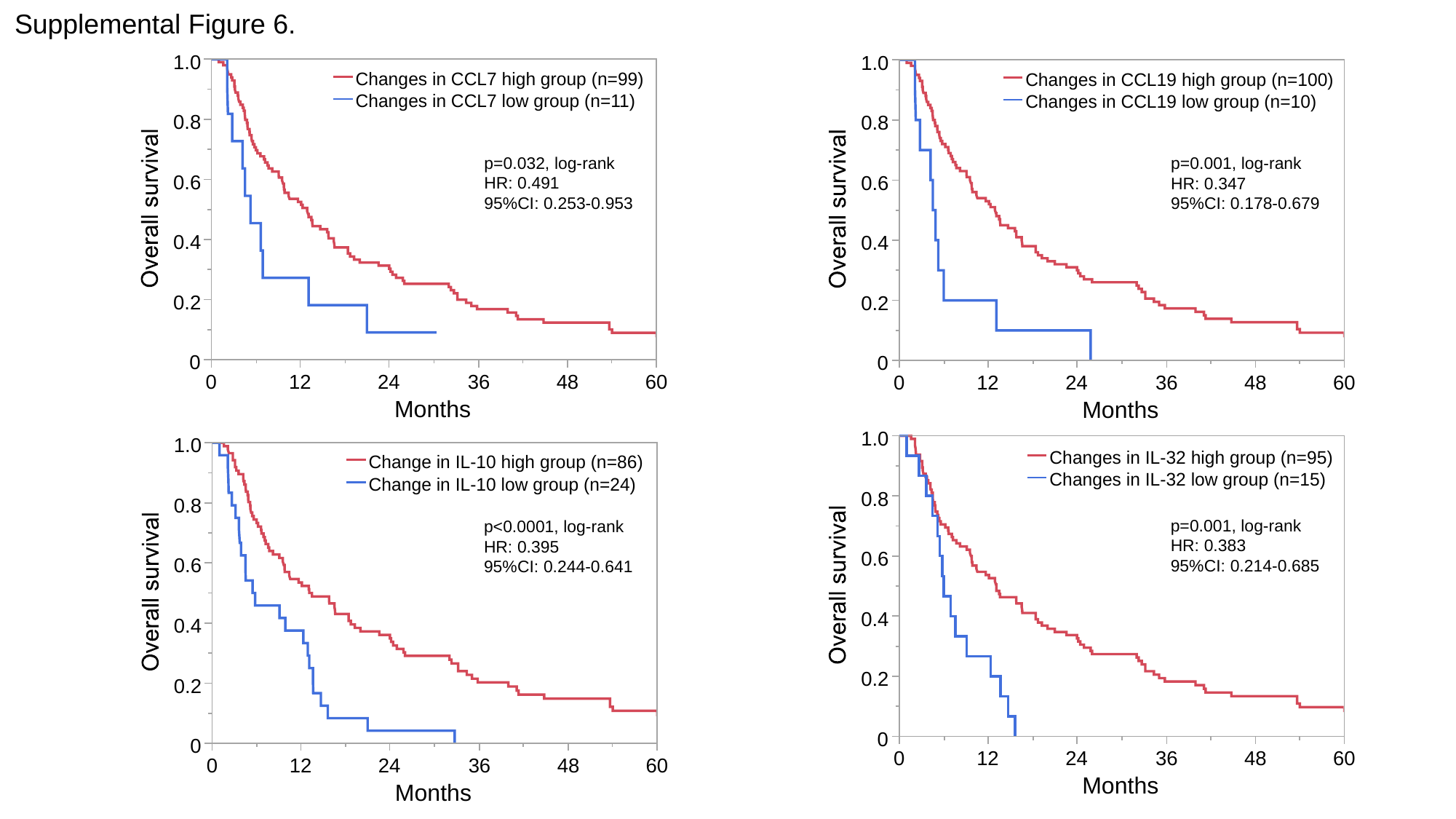

Supplemental Figure 6.
p=0.032, log-rank
HR: 0.491
95%CI: 0.253-0.953
p=0.001, log-rank
HR: 0.347
95%CI: 0.178-0.679
p=0.001, log-rank
HR: 0.383
95%CI: 0.214-0.685
p<0.0001, log-rank
HR: 0.395
95%CI: 0.244-0.641

## Slide 7
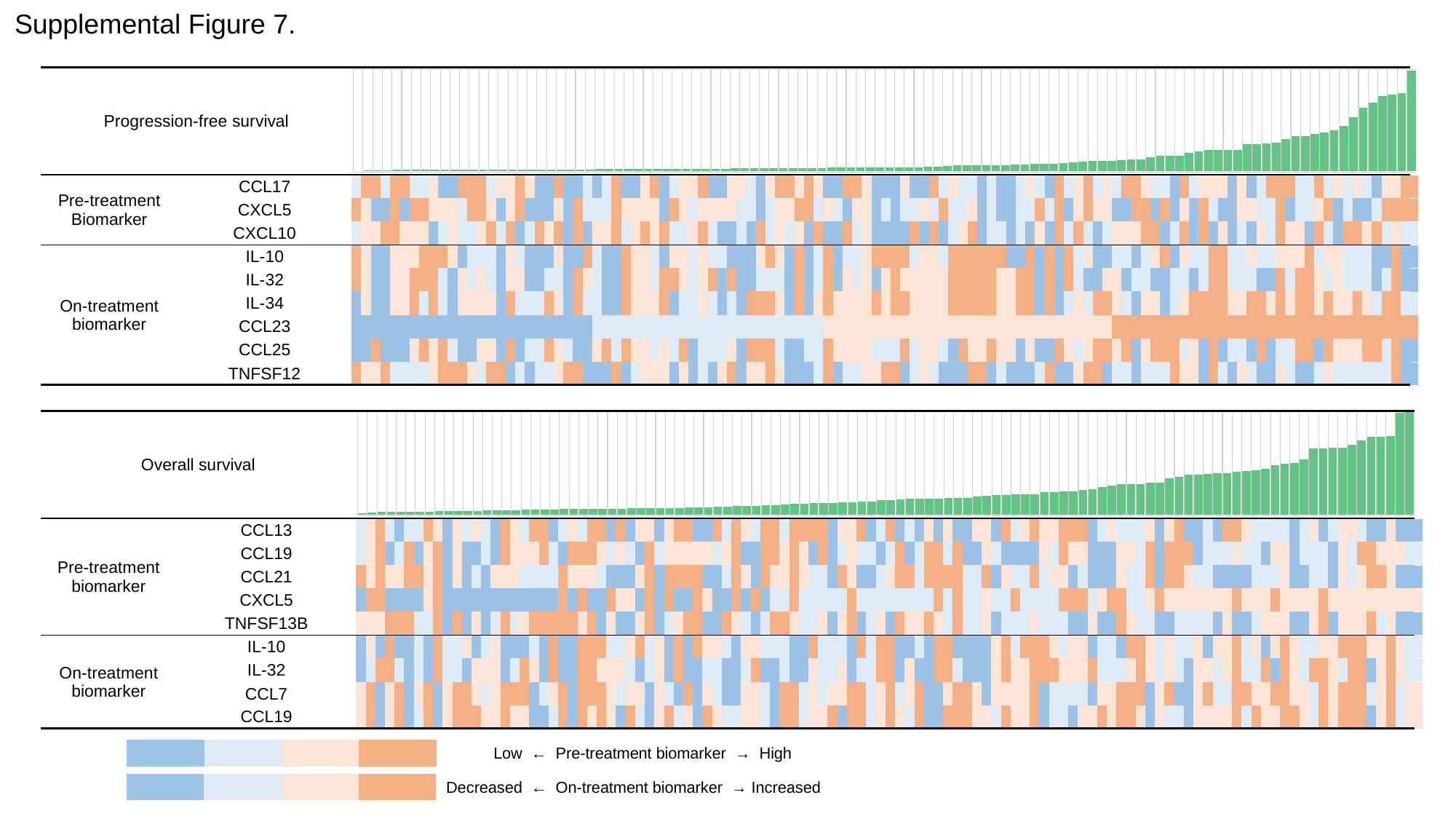

Supplemental Figure 7.
| Progression-free survival | | | | | | | | | | | | | | | | | | | | | | | | | | | | | | | | | | | | | | | | | | | | | | | | | | | | | | | | | | | | | | | | | | | | | | | | | | | | | | | | | | | | | | | | | | | | | | | | | | | | | | | | | | | | | | | |
| --- | --- | --- | --- | --- | --- | --- | --- | --- | --- | --- | --- | --- | --- | --- | --- | --- | --- | --- | --- | --- | --- | --- | --- | --- | --- | --- | --- | --- | --- | --- | --- | --- | --- | --- | --- | --- | --- | --- | --- | --- | --- | --- | --- | --- | --- | --- | --- | --- | --- | --- | --- | --- | --- | --- | --- | --- | --- | --- | --- | --- | --- | --- | --- | --- | --- | --- | --- | --- | --- | --- | --- | --- | --- | --- | --- | --- | --- | --- | --- | --- | --- | --- | --- | --- | --- | --- | --- | --- | --- | --- | --- | --- | --- | --- | --- | --- | --- | --- | --- | --- | --- | --- | --- | --- | --- | --- | --- | --- | --- | --- | --- |
| Pre-treatment Biomarker | CCL17 | | | | | | | | | | | | | | | | | | | | | | | | | | | | | | | | | | | | | | | | | | | | | | | | | | | | | | | | | | | | | | | | | | | | | | | | | | | | | | | | | | | | | | | | | | | | | | | | | | | | | | | | | | | | | | |
| | CXCL5 | | | | | | | | | | | | | | | | | | | | | | | | | | | | | | | | | | | | | | | | | | | | | | | | | | | | | | | | | | | | | | | | | | | | | | | | | | | | | | | | | | | | | | | | | | | | | | | | | | | | | | | | | | | | | | |
| | CXCL10 | | | | | | | | | | | | | | | | | | | | | | | | | | | | | | | | | | | | | | | | | | | | | | | | | | | | | | | | | | | | | | | | | | | | | | | | | | | | | | | | | | | | | | | | | | | | | | | | | | | | | | | | | | | | | | |
| On-treatment biomarker | IL-10 | | | | | | | | | | | | | | | | | | | | | | | | | | | | | | | | | | | | | | | | | | | | | | | | | | | | | | | | | | | | | | | | | | | | | | | | | | | | | | | | | | | | | | | | | | | | | | | | | | | | | | | | | | | | | | |
| | IL-32 | | | | | | | | | | | | | | | | | | | | | | | | | | | | | | | | | | | | | | | | | | | | | | | | | | | | | | | | | | | | | | | | | | | | | | | | | | | | | | | | | | | | | | | | | | | | | | | | | | | | | | | | | | | | | | |
| | IL-34 | | | | | | | | | | | | | | | | | | | | | | | | | | | | | | | | | | | | | | | | | | | | | | | | | | | | | | | | | | | | | | | | | | | | | | | | | | | | | | | | | | | | | | | | | | | | | | | | | | | | | | | | | | | | | | |
| | CCL23 | | | | | | | | | | | | | | | | | | | | | | | | | | | | | | | | | | | | | | | | | | | | | | | | | | | | | | | | | | | | | | | | | | | | | | | | | | | | | | | | | | | | | | | | | | | | | | | | | | | | | | | | | | | | | | |
| | CCL25 | | | | | | | | | | | | | | | | | | | | | | | | | | | | | | | | | | | | | | | | | | | | | | | | | | | | | | | | | | | | | | | | | | | | | | | | | | | | | | | | | | | | | | | | | | | | | | | | | | | | | | | | | | | | | | |
| | TNFSF12 | | | | | | | | | | | | | | | | | | | | | | | | | | | | | | | | | | | | | | | | | | | | | | | | | | | | | | | | | | | | | | | | | | | | | | | | | | | | | | | | | | | | | | | | | | | | | | | | | | | | | | | | | | | | | | |
| Overall survival | | | | | | | | | | | | | | | | | | | | | | | | | | | | | | | | | | | | | | | | | | | | | | | | | | | | | | | | | | | | | | | | | | | | | | | | | | | | | | | | | | | | | | | | | | | | | | | | | | | | | | | | | | | | | | | |
| --- | --- | --- | --- | --- | --- | --- | --- | --- | --- | --- | --- | --- | --- | --- | --- | --- | --- | --- | --- | --- | --- | --- | --- | --- | --- | --- | --- | --- | --- | --- | --- | --- | --- | --- | --- | --- | --- | --- | --- | --- | --- | --- | --- | --- | --- | --- | --- | --- | --- | --- | --- | --- | --- | --- | --- | --- | --- | --- | --- | --- | --- | --- | --- | --- | --- | --- | --- | --- | --- | --- | --- | --- | --- | --- | --- | --- | --- | --- | --- | --- | --- | --- | --- | --- | --- | --- | --- | --- | --- | --- | --- | --- | --- | --- | --- | --- | --- | --- | --- | --- | --- | --- | --- | --- | --- | --- | --- | --- | --- | --- | --- |
| Pre-treatment biomarker | CCL13 | | | | | | | | | | | | | | | | | | | | | | | | | | | | | | | | | | | | | | | | | | | | | | | | | | | | | | | | | | | | | | | | | | | | | | | | | | | | | | | | | | | | | | | | | | | | | | | | | | | | | | | | | | | | | | |
| | CCL19 | | | | | | | | | | | | | | | | | | | | | | | | | | | | | | | | | | | | | | | | | | | | | | | | | | | | | | | | | | | | | | | | | | | | | | | | | | | | | | | | | | | | | | | | | | | | | | | | | | | | | | | | | | | | | | |
| | CCL21 | | | | | | | | | | | | | | | | | | | | | | | | | | | | | | | | | | | | | | | | | | | | | | | | | | | | | | | | | | | | | | | | | | | | | | | | | | | | | | | | | | | | | | | | | | | | | | | | | | | | | | | | | | | | | | |
| | CXCL5 | | | | | | | | | | | | | | | | | | | | | | | | | | | | | | | | | | | | | | | | | | | | | | | | | | | | | | | | | | | | | | | | | | | | | | | | | | | | | | | | | | | | | | | | | | | | | | | | | | | | | | | | | | | | | | |
| | TNFSF13B | | | | | | | | | | | | | | | | | | | | | | | | | | | | | | | | | | | | | | | | | | | | | | | | | | | | | | | | | | | | | | | | | | | | | | | | | | | | | | | | | | | | | | | | | | | | | | | | | | | | | | | | | | | | | | |
| On-treatment biomarker | IL-10 | | | | | | | | | | | | | | | | | | | | | | | | | | | | | | | | | | | | | | | | | | | | | | | | | | | | | | | | | | | | | | | | | | | | | | | | | | | | | | | | | | | | | | | | | | | | | | | | | | | | | | | | | | | | | | |
| | IL-32 | | | | | | | | | | | | | | | | | | | | | | | | | | | | | | | | | | | | | | | | | | | | | | | | | | | | | | | | | | | | | | | | | | | | | | | | | | | | | | | | | | | | | | | | | | | | | | | | | | | | | | | | | | | | | | |
| | CCL7 | | | | | | | | | | | | | | | | | | | | | | | | | | | | | | | | | | | | | | | | | | | | | | | | | | | | | | | | | | | | | | | | | | | | | | | | | | | | | | | | | | | | | | | | | | | | | | | | | | | | | | | | | | | | | | |
| | CCL19 | | | | | | | | | | | | | | | | | | | | | | | | | | | | | | | | | | | | | | | | | | | | | | | | | | | | | | | | | | | | | | | | | | | | | | | | | | | | | | | | | | | | | | | | | | | | | | | | | | | | | | | | | | | | | | |
| | | | | Low ← Pre-treatment biomarker → High |
| --- | --- | --- | --- | --- |
| | | | | Decreased ← On-treatment biomarker → Increased |
| --- | --- | --- | --- | --- |

## Slide 8
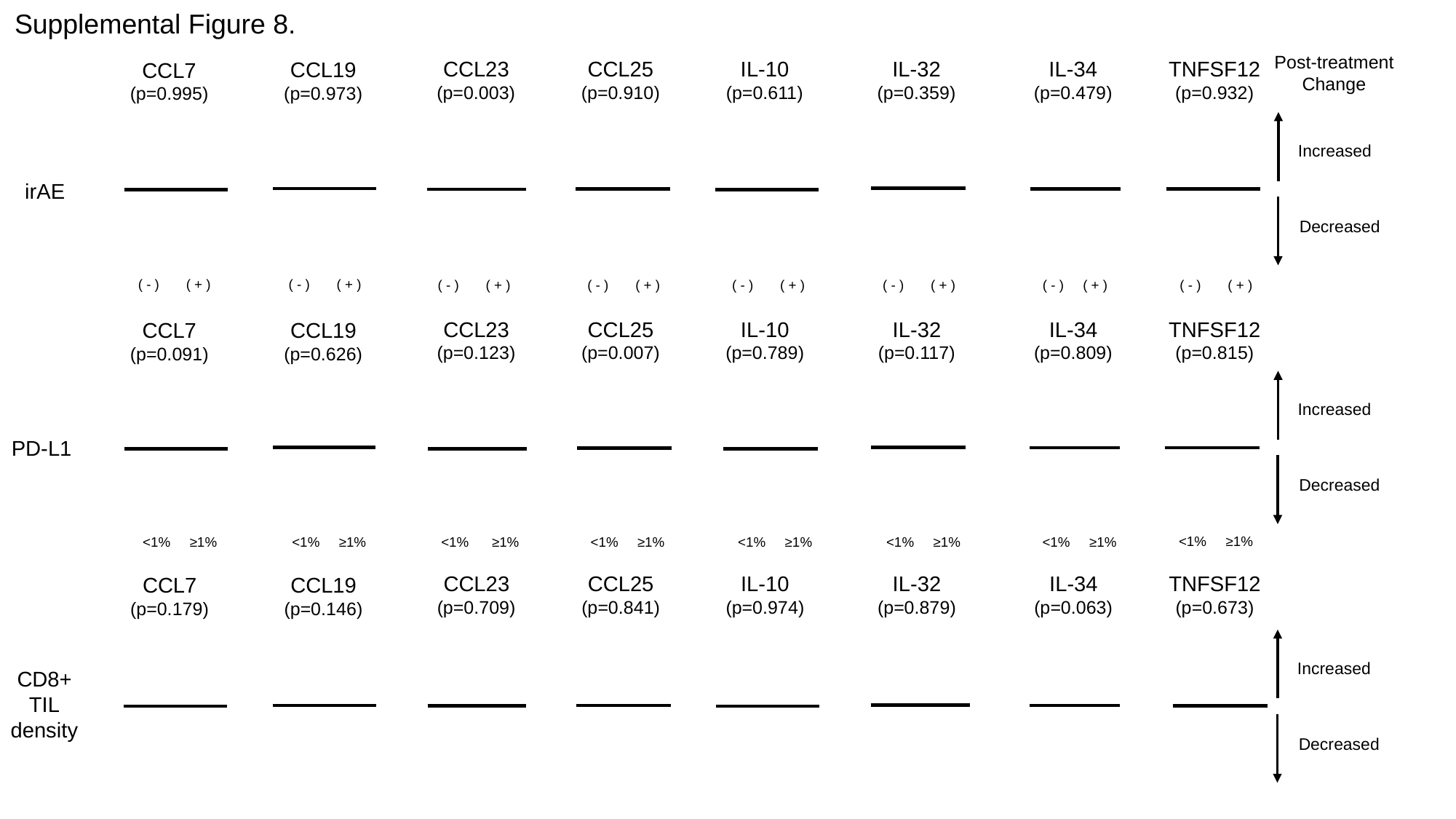

Supplemental Figure 8.
Post-treatment
Change
CCL23
(p=0.003)
CCL25
(p=0.910)
IL-10
(p=0.611)
IL-32
(p=0.359)
IL-34
(p=0.479)
TNFSF12
(p=0.932)
CCL19
(p=0.973)
CCL7
(p=0.995)
Increased
irAE
Decreased
( - ) ( + )
( - ) ( + )
( - ) ( + )
( - ) ( + )
( - ) ( + )
( - ) ( + )
( - ) ( + )
( - ) ( + )
CCL23
(p=0.123)
CCL25
(p=0.007)
IL-10
(p=0.789)
IL-32
(p=0.117)
IL-34
(p=0.809)
TNFSF12
(p=0.815)
CCL19
(p=0.626)
CCL7
(p=0.091)
Increased
PD-L1
Decreased
<1% ≥1%
<1% ≥1%
<1% ≥1%
<1% ≥1%
<1% ≥1%
<1% ≥1%
<1% ≥1%
<1% ≥1%
CCL23
(p=0.709)
CCL25
(p=0.841)
IL-10
(p=0.974)
IL-32
(p=0.879)
IL-34
(p=0.063)
TNFSF12
(p=0.673)
CCL19
(p=0.146)
CCL7
(p=0.179)
Increased
CD8+
TIL
density
Decreased

## Slide 9
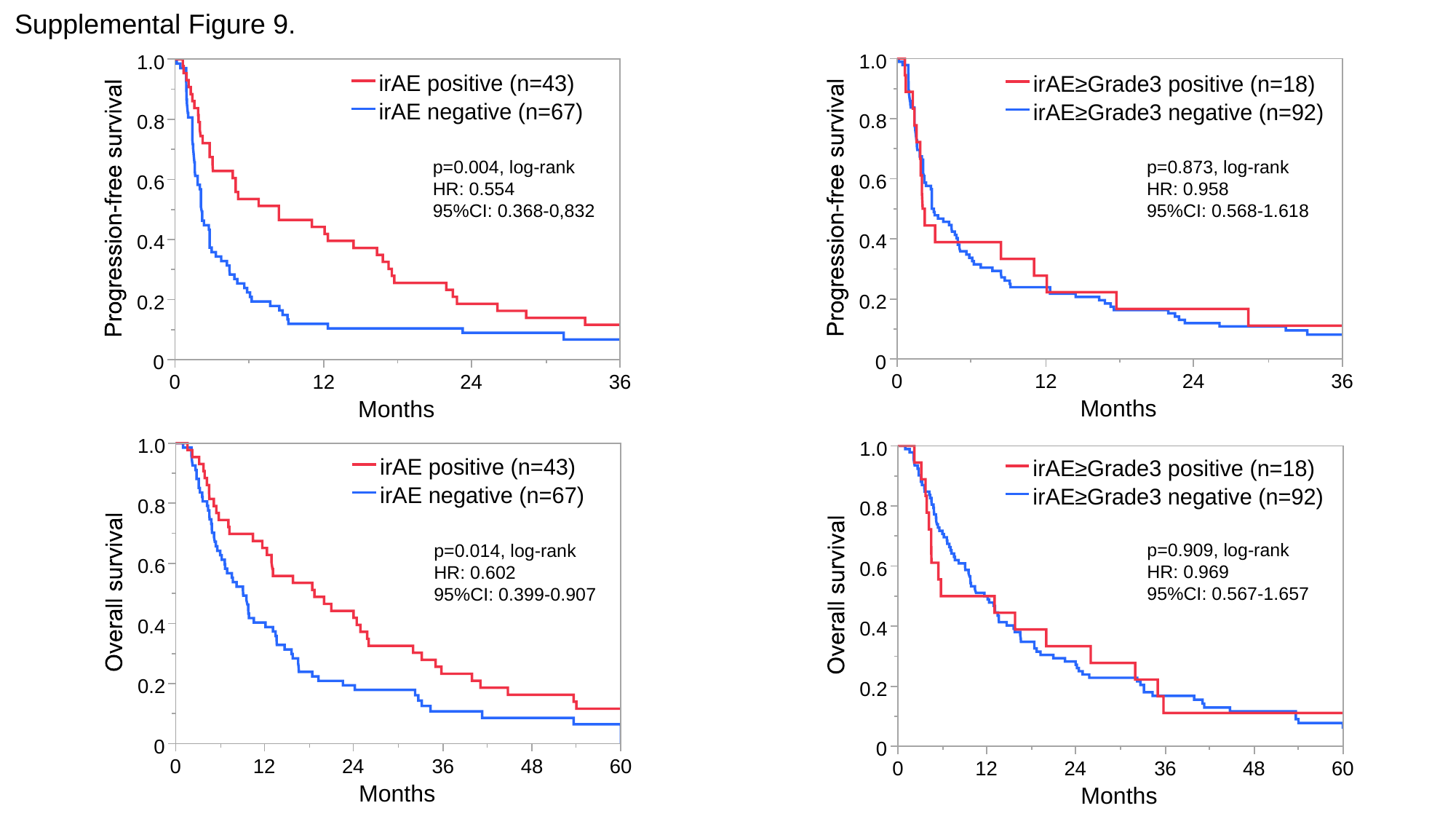

Supplemental Figure 9.
p=0.873, log-rank
HR: 0.958
95%CI: 0.568-1.618
p=0.004, log-rank
HR: 0.554
95%CI: 0.368-0,832
p=0.909, log-rank
HR: 0.969
95%CI: 0.567-1.657
p=0.014, log-rank
HR: 0.602
95%CI: 0.399-0.907

## Slide 10
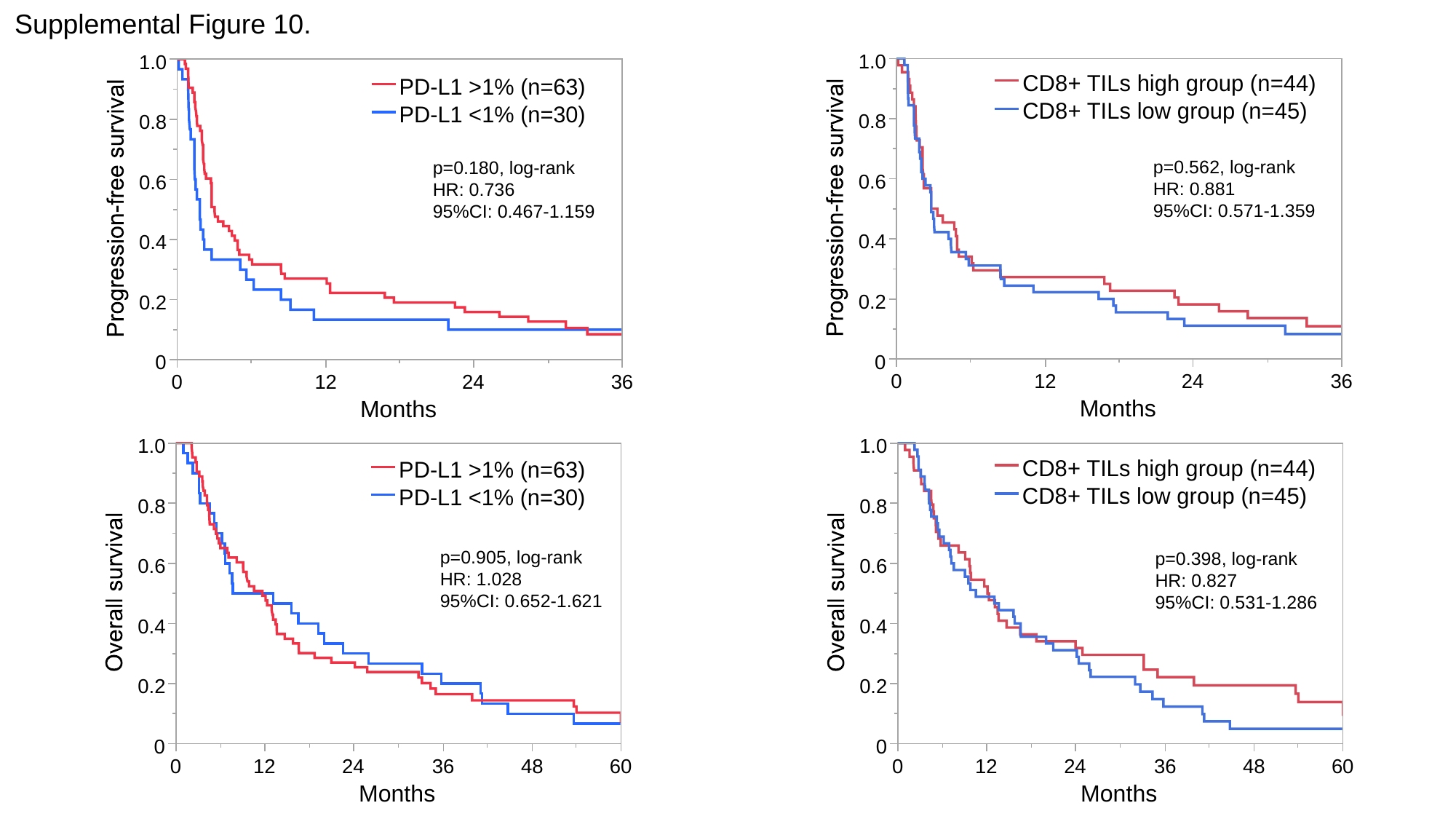

Supplemental Figure 10.
p=0.562, log-rank
HR: 0.881
95%CI: 0.571-1.359
p=0.180, log-rank
HR: 0.736
95%CI: 0.467-1.159
p=0.905, log-rank
HR: 1.028
95%CI: 0.652-1.621
p=0.398, log-rank
HR: 0.827
95%CI: 0.531-1.286

## Slide 11
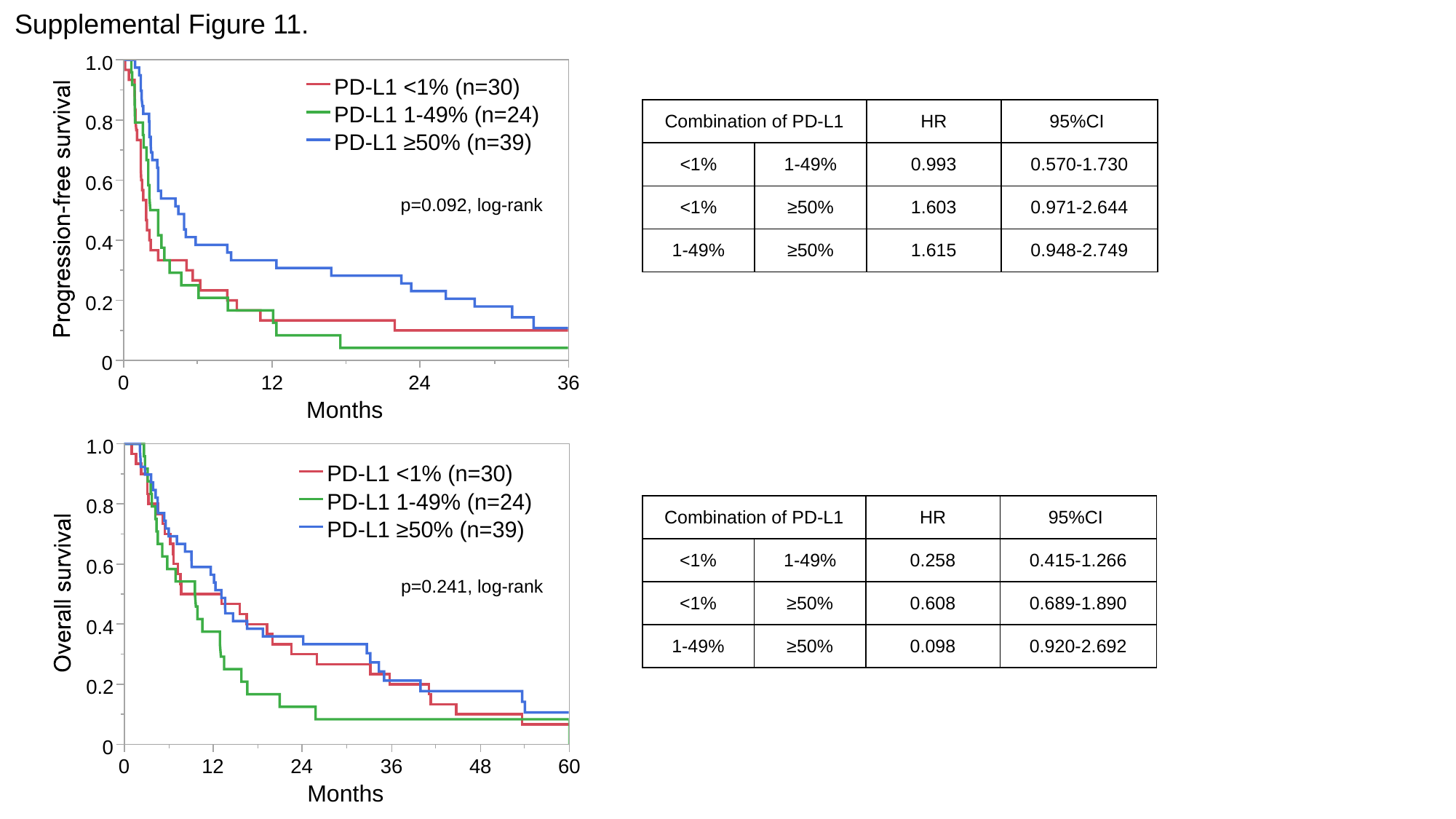

Supplemental Figure 11.
| Combination of PD-L1 | | HR | 95%CI |
| --- | --- | --- | --- |
| <1% | 1-49% | 0.993 | 0.570-1.730 |
| <1% | ≥50% | 1.603 | 0.971-2.644 |
| 1-49% | ≥50% | 1.615 | 0.948-2.749 |
p=0.092, log-rank
| Combination of PD-L1 | | HR | 95%CI |
| --- | --- | --- | --- |
| <1% | 1-49% | 0.258 | 0.415-1.266 |
| <1% | ≥50% | 0.608 | 0.689-1.890 |
| 1-49% | ≥50% | 0.098 | 0.920-2.692 |
p=0.241, log-rank

## Slide 12
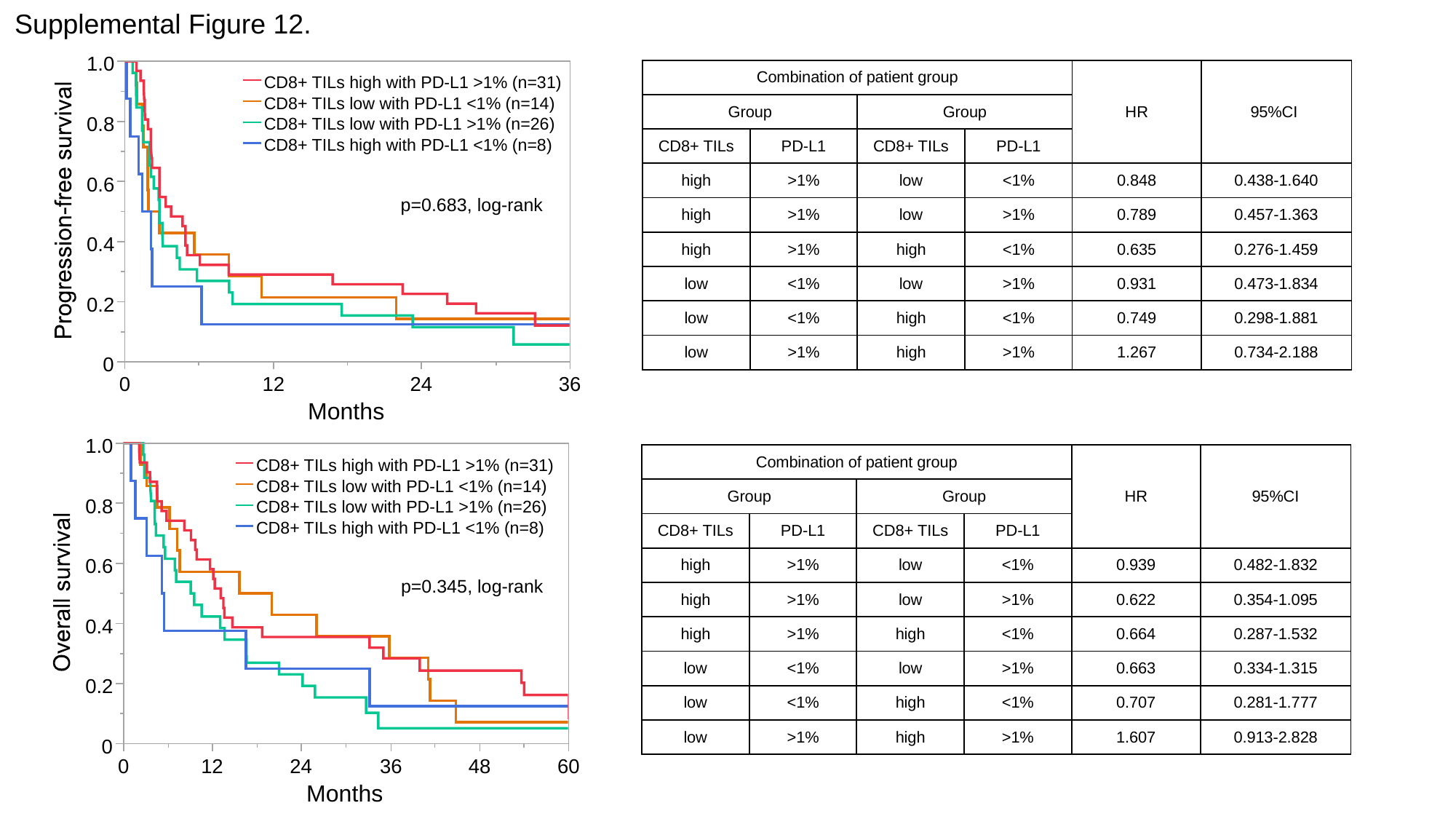

Supplemental Figure 12.
| Combination of patient group | | | | HR | 95%CI |
| --- | --- | --- | --- | --- | --- |
| Group | | Group | | | |
| CD8+ TILs | PD-L1 | CD8+ TILs | PD-L1 | | |
| high | >1% | low | <1% | 0.848 | 0.438-1.640 |
| high | >1% | low | >1% | 0.789 | 0.457-1.363 |
| high | >1% | high | <1% | 0.635 | 0.276-1.459 |
| low | <1% | low | >1% | 0.931 | 0.473-1.834 |
| low | <1% | high | <1% | 0.749 | 0.298-1.881 |
| low | >1% | high | >1% | 1.267 | 0.734-2.188 |
p=0.683, log-rank
| Combination of patient group | | | | HR | 95%CI |
| --- | --- | --- | --- | --- | --- |
| Group | | Group | | | |
| CD8+ TILs | PD-L1 | CD8+ TILs | PD-L1 | | |
| high | >1% | low | <1% | 0.939 | 0.482-1.832 |
| high | >1% | low | >1% | 0.622 | 0.354-1.095 |
| high | >1% | high | <1% | 0.664 | 0.287-1.532 |
| low | <1% | low | >1% | 0.663 | 0.334-1.315 |
| low | <1% | high | <1% | 0.707 | 0.281-1.777 |
| low | >1% | high | >1% | 1.607 | 0.913-2.828 |
p=0.345, log-rank
