# Supplementary material for: Characterization of pre- and on-treatment soluble immune mediators and the tumor microenvironment in NSCLC patients receiving PD-1/L1 inhibitor monotherapy
Source: Cancer Immunol Immunother. 2024 Sep 5;73(11):214. doi: 10.1007/s00262-024-03781-8 (PMC11377373; doi:10.1007/s00262-024-03781-8)
Supplement: Supplementary file 3 — Supplementary file3 (DOCX 12 kb) [file 262_2024_3781_MOESM3_ESM.docx]

Supplemental appendix. Measured soluble immune mediators

We measured the following soluble immune mediators: CCL1, CCL2, CCL3, CCL7, CCL8, CCL11, CCL13, CCL15, CCL17, CCL19, CCL20, CCL21, CCL22, CCL23, CCL24, CCL25, CCL26, CCL27, Chitinase-3-like-1, CX3CL1, CXCL1, CXCL2, CXCL5, CXCL6, CXCL8, CXCL9, CXCL10, CXCL11, CXCL12, CXCL13, CXCL16, GM-CSF, IFN-α2, IFN-β, IFN-γ, IL-1β, IL-2, IL-4, IL-6, IL-10, IL-11, IL-12, IL-12(p70), IL-16, IL-19, IL-20, IL-22, IL-26, IL-27, IL-29, IL-32, IL-34, IL-35, IL-6Ra, MIF, MMP-1, MMP-2, MMP-3, Osteocalcin, Osteopontin, Pentraxin-3, TNF-α, sCD163, sIL-6Rb, sTNF-R1, sTNF-R2, TNFRSF8, TNFSF12, TNFSF13, TNFSF13B, TNFSF14, TSLP and VEGF.
